# Multipoint Costriking Nanodevice Eliminates Primary Tumor Cells and Associated‐Circulating Tumor Cells for Enhancing Metastasis Inhibition and Therapeutic Effect on HCC

**DOI:** 10.1002/advs.202101472

**Published:** 2022-01-14

**Authors:** Weiwei Mu, Qihui Chu, Huizhen Yang, Li Guan, Shunli Fu, Tong Gao, Xiao Sang, Zipeng Zhang, Shuang Liang, Yongjun Liu, Na Zhang

**Affiliations:** ^1^ Department of Pharmaceutics Key Laboratory of Chemical Biology (Ministry of Education) School of Pharmaceutical Sciences Cheeloo College of Medicine Shandong University 44 Wenhuaxi Road Jinan Shandong Province 250012 China

**Keywords:** circulating tumor cells, CTC clusters, CTC–neutrophil clusters, hepatocellular carcinoma, metastasis inhibition, multipoint costriking nanodevice

## Abstract

Eliminating primary tumor (“roots”) and inhibiting associated‐circulating tumor cells (associated‐CTCs, “seeds”) are vital issues that need to be urgently addressed in cancer therapy. Associated‐CTCs, which include single CTCs, CTC clusters, and CTC–neutrophil clusters, are essential executors in metastasis and the cause of metastasis‐related death in cancer patients. Herein, a “roots and seeds” multipoint costriking nanodevice (GV‐Lipo/sorafenib (SF)/digitoxin (DT)) is developed to eliminate primary tumors and inhibit the spread of associated‐CTCs for enhancing metastasis inhibition and the therapeutic effect on hepatocellular carcinoma (HCC). GV‐Lipo/SF/DT eliminates primary tumor cells by the action of SF, thus reducing CTC production at the roots and improving the therapeutic effect on HCC. GV‐Lipo/SF/DT inhibits associated‐CTCs effectively via the enhanced identification and capture effects of glypican‐3 and/or vascular cell adhesion molecule 1 (VCAM1) targeting, dissociating CTC clusters using DT, blocking the formation of CTC–neutrophil clusters using anti‐VCAM1 monoclonal antibody, and killing CTCs with SF. It is successfully verified that GV‐Lipo/SF/DT increases the CTC elimination efficiency in vivo, thus effectively preventing metastasis, and shows enhanced antitumor efficacy in both an H22‐bearing tumor model and orthotopic HCC models. Overall, the “roots and seeds” multipoint costriking strategy may open a new cancer treatment model for the clinic.

## Graphical abstract

A multipoint costriking nanodevice (GV‐Lipo/SF/DT) with the capability of eliminates primary tumor cells and associated‐circulating tumor cells (CTCs) is obtained for enhancing metastasis inhibition and therapeutic effect on HCC. GV‐Lipo/SF/DT can specifically target tumor cells and capture CTCs, dissociate CTC clusters, block the formation of CTC–neutrophil clusters, finally kill CTCs and primary tumor cells.

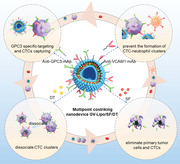

## 1 Introduction

Metastasis is the leading cause of death in cancer patients.^[^ ^1^ ^]^ Metastasis is a multistep cellular biological process in which primary tumor cells extravasate into the bloodstream and survive as circulating tumor cells (CTCs).^[^ ^2^, ^3^ ^]^ Then, CTCs extravasate into surrounding organs and form metastasis initiating cells or metastatic nodes.^[^ ^4^ ^]^ Primary tumor cells can be considered the “roots” of metastasis, while CTCs can be considered the “seeds” of metastasis, and together they confer a poor prognosis for cancer patients. Therefore, both the elimination of “roots” and the inhibition of the spread of “seeds” are vital issues that urgently need to be addressed in cancer treatment. Conventional treatments, such as surgical treatment, chemotherapy, and immunotherapy, have made great progress in eliminating primary tumors,^[^ ^5^, ^6^, ^7^ ^]^ but metastasis and recurrence after treatment still lead to a low survival rate. Currently, some strategies have focused on the importance of CTCs.^[^ ^8^, ^9^ ^]^ For example, an exosome‐like sequential‐bioactivating prodrug nanoplatform was designed for breast cancer therapy and found to suppress metastasis through CTC clearance.^[^ ^10^ ^]^

With the in‐depth study of CTCs, it has been found that in addition to single CTCs, CTC clusters and CTC–neutrophil clusters are present in the bloodstream of cancer patients and have a high potential to promote tumor metastasis.^[^ ^11^, ^12^ ^]^ CTC clusters are groupings of multiple cells derived from primary tumor cells and are more efficient in forming metastases than single CTCs.^[^ ^13^, ^14^ ^]^ CTC clusters form strong cell‐cell adhesion through desmosomes and adherens junctions, which could help overcome anoikis and promote metastasis.^[^ ^15^ ^]^ The dissociation of CTC clusters remodels key binding sites and suppresses metastasis. CTCs can also associate with neutrophils to form CTC–neutrophil clusters and maintain high levels of proliferation in the circulation, and the adhesion of CTCs to neutrophils promotes extravasation and metastasis.^[^ ^16^, ^17^ ^]^ Increasing evidence has shown that neutrophils interact with CTCs via binding to highly expressed adhesion molecules on tumor cells, such as vascular cell adhesion molecule 1 (VCAM1).^[^ ^18^, ^19^ ^]^ Inhibition of the VCAM1 binding prevents the formation of CTC–neutrophil clusters and thus inhibits tumor metastasis.^[^ ^17^ ^]^ CTC clusters and CTC–neutrophil clusters show increased metastatic potential compared with single CTCs and are as important as single CTCs in the process of tumor metastasis. Here, single CTCs, CTC clusters, and CTC–neutrophil clusters are collectively considered associated‐CTCs. Although CTCs have become one of the critical targets for cancer therapy, an effective therapeutic system focusing on primary tumor cells and associated‐CTCs simultaneously has not been developed to date. Therefore, designing a “roots and seeds” multipoint costriking strategy that could simultaneously eliminate primary tumor cells and prevent metastasis by targeting associated‐CTCs would be a promising strategy to improve the treatment effect on cancer.

Hepatocellular carcinoma (HCC) has a high metastatic potential and the third highest fatality rate among cancers worldwide; thus, effective therapeutic strategies are urgently needed.^[^ ^20^, ^21^ ^]^ To implement the “roots and seeds” multipoint costriking strategy to treat HCC, it is necessary to improve the efficiency of CTC identification and capture. CTCs are extremely rare in the bloodstream, and a more specific and sensitive CTC‐targeting capture system is urgently needed.^[^ ^22^, ^23^, ^24^ ^]^ Glypican‐3 (GPC3) is an oncofetal proteoglycan with high specific expression in HCC that possesses great potential for early diagnosis and precise therapy of HCC.^[^ ^25^, ^26^ ^]^ In our previous work, it was indicated that GPC3 targeting could realize early and high‐sensitivity theranostics in HCC.^[^ ^27^ ^]^ Thus, it is hypothesized that GPC3 is a reliable target for the identification and capture of associated‐CTCs in HCC. It was proven that Na^+^/K^+^ ATPase inhibitors could induce loss of cell–cell junctions in cancer cells by increasing intracellular Ca^2+^ levels, which dissociated CTC clusters into single CTCs and suppressed metastasis formation.^[^ ^28^ ^]^ Digitoxin (DT) is an effective Na^+^/K^+^ ATPase inhibitor approved by the FDA and has a great capacity to maximize the dissociation of CTC clusters.^[^ ^11^ ^]^ Thus, DT possesses relatively good clinical prospects for CTC cluster dissociation. Using an anti‐VCAM1 monoclonal antibody (mAb) to bind VCAM1, which is overexpressed on the membrane of CTCs, would prevent the formation of CTC–neutrophil clusters. Moreover, anti‐VCAM1 mAb and anti‐GPC3 mAb could specifically recognize and combine with their receptors overexpressed on the membrane of CTC respectively improving the targeting sensitivity of therapies directed against CTCs and primary tumor cells.

**Scheme 1:**
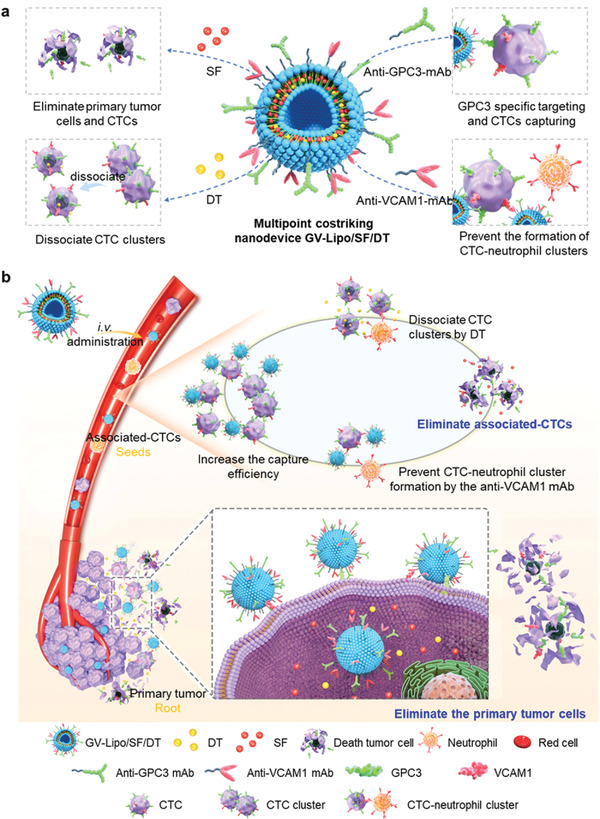
Illustration of the multipoint costriking nanodevice (GV‐Lipo/SF/DT) eliminates primary tumor and associated‐CTCs for enhancing metastasis inhibition and therapeutic effect on HCC. a) Composition of the GV‐Lipo/SF/DT. GV‐Lipo/SF/DT were developed by coloading SF and DT into liposomes, and modifying anti‐GPC3 mAb and anti‐VCAM1 mAb on the surface of the coloaded liposomes; b) GV‐Lipo/SF/DT eliminates primary tumor cells through increased tumor cell recognition and uptake via GPC3 and/or VCAM1 targeting, kills primary tumor cells by SF, reduces CTC production and improves the therapeutic effect on HCC. GV‐Lipo/SF/DT eliminates associated‐CTCs in the bloodstream by specific targeting that led to recognition and capture of associated‐CTCs via GPC3 and/or VCAM1 targeting, dissociation of CTC clusters using DT, prevention CTC–neutrophil cluster formation by the anti‐VCAM1 mAb and elimination of CTCs by SF.

Herein, we designed a primary tumor and associated‐CTCs multipoint costriking nanodevice (GV‐Lipo/SF/DT) for enhancing metastasis inhibition and therapeutic effect on HCC. GV‐Lipo/SF/DT was developed by coloading sorafenib (SF) and DT into liposomes, and modifying anti‐GPC3 mAb and anti‐VCAM1 mAb on the surface of the coloaded liposomes (**Scheme** **1**a). SF, the first multikinase inhibitor approved by the FDA for first‐line treatment of HCC, was selected as the chemotherapeutic drug to eliminate the “roots and seeds”. GV‐Lipo/SF/DT could eliminate primary tumor cells by the action of SF and prevent metastasis through specific targeting that recognized and captured associated‐CTCs via GPC3 and/or VCAM1 targeting, dissociating CTC clusters using DT, preventing CTC–neutrophil cluster formation with the anti‐VCAM1 mAb and eliminating CTCs by SF (Scheme 1b). We successfully verified that GV‐Lipo/SF/DT possessed an increased targeting ability and enhanced CTC elimination efficiency in vivo. Enhanced antitumor efficacy was observed in both H22‐bearing HCC models and orthotopic HCC models. Furthermore, GV‐Lipo/SF/DT effectively prevented metastasis by inhibiting associated‐CTCs. To the best of our knowledge, the “roots and seeds” multipoint costriking strategy is the first attempt to combine primary tumor cells and associated‐CTCs for cancer therapy and metastasis inhibition, which will provide a theoretical and experimental foundation for improving therapeutic effects on various cancers.

## 2 Results

### 2.1 Characterization and Enhanced Targeting Ability of GV‐Lipo/SF/DT

**Figure 1:**
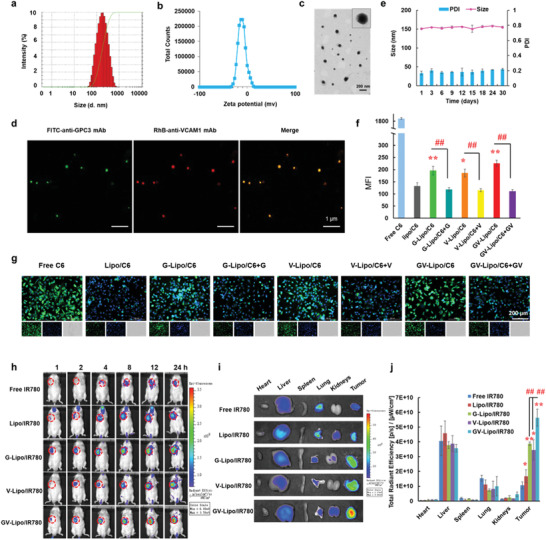
GV‐Lipo/SF/DT showed a specific targeting ability. a) Particle size, b) zeta potential, and c) transmission electron microscopy (TEM) imaging of GV‐Lipo/SF/DT. d) CLSM images of FITC and RhB double labeled GV‐Lipo/SF/DT. Anti‐GPC3 mAb was labeled with FITC and anti‐VCAM1 mAb was labeled with RhB. e) The storage stability of GV‐Lipo/SF/DT at 4 °C for 30 days. f–g) Cellular uptake and competitive inhibition experiments performed with Hepa1‐6 cells and free C6, Lipo/C6, G‐Lipo/C6, V‐Lipo/C6 or GV‐Lipo/C6. (f) Quantification of fluorescence intensity by FCM. *n* = 3, *p < 0.05, and **p < 0.01 compared to Lipo/C6, ^##^ *p* < 0.01. (g) Inverted fluorescence microscopy images of different groups. h) NIRF imaging of H22 tumor‐bearing mice after i.v. injection of different formulations (free IR780, Lipo/IR780, G‐Lipo/IR780, V‐Lipo/IR780, or GV‐Lipo/IR780; IR780 dose: 1 mg kg^−1^) at 1, 2, 4, 8, 12, and 24 h. Tumors are highlighted in red circles. i) Representative images of the heart, liver, spleen, lung, kidneys, and tumor in each group at 24 h postadministration. j) Total radiant efficiency in the main heart, liver, spleen, lungs, kidneys, and tumor based on the ex vivo results. *p < 0.05 and **p < 0.01 compared to free IR780, ^#^ *p* < 0.05, ^##^ *p* < 0.01.

The multipoint costriking nanodevice GV‐Lipo/SF/DT was prepared by coloading SF and DT into liposomes, and then the surface of the coloaded liposomes was dual modified with anti‐GPC3 mAb and anti‐VCAM1 mAb through DSPE‐PEG_2000_. First, the DSPE‐PEG_2000_‐anti‐GPC3 mAb and DSPE‐PEG_2000_‐anti‐VCAM1 mAb were successfully synthesized and characterized by ^1^H nuclear magnetic resonance (^1^H‐NMR) (Figure S1, Supporting Information). The synthetic yield of DSPE‐PEG_2000_‐anti‐GPC3 mAb was 78.86% ± 2.24% and the synthetic yield of DSPE‐PEG_2000_‐anti‐VCAM1 mAb was 78.28% ± 5.75%. Then, the DSPE‐PEG_2000_‐anti‐GPC3 mAb and DSPE‐PEG_2000_‐anti‐VCAM1 mAb were both conjugated to the surface of liposomes with modification rates of 99.38% and 99.32%, respectively. GV‐Lipo/SF/DT comprised well‐dispersed spheres with a particle size of 148.77 ± 5.88 nm and zeta potential of −11.20 ± 0.46 mV (**Figure** **1**a–c). The particle size and zeta potential of Lipo/SF/DT was 126.13 ± 3.13 nm and −13.63 ± 0.25 mV, respectively (Figure S2, Table S1, Supporting Information). The encapsulation efficiency (EE%) of GV‐Lipo/SF/DT was 84.38% ± 4.56% for SF and 89.07% ± 1.69% for DT (Table S1, Supporting Information). To visualize the successful modification of liposomes with the anti‐GPC3 mAb and anti‐VCAM1 mAb, we used FITC to label the anti‐GPC3 mAb and rhodamine B (RhB) to label the anti‐VCAM1 mAb. Confocal laser scanning microscopy (CLSM) imaging of FITC and RhB double‐labeled GV‐Lipo/SF/DT indicated that the DSPE‐PEG_2000_‐anti‐GPC3 mAb and DSPE‐PEG_2000_‐anti‐VCAM1 mAb were successfully conjugated to the surface of liposomes (Figure 1d). Additionally, GV‐Lipo/SF/DT showed the desired storage stability over 30 days at 4 °C (Figure 1e) and physical stability in 20% plasma for 72 h (Figure S3, Supporting Information). Furthermore, the in vitro cumulative release of SF from GV‐Lipo/SF/DT was 58.84% ± 5.21% over 72 h (Figure S4, Supporting Information), and that of DT from GV‐Lipo/SF/DT was 98.73% ± 0.83% (Figure S5, Supporting Information). GV‐Lipo/SF/DT showed better cytotoxicity to Hepa1‐6 cells than Lipo/SF/DT (Figure S6, Table S2, Supporting Information).

Both the Hepa1‐6 cell line and the H22 cell line were selected as mouse‐derived HCC cell models. We evaluated the expression of GPC3 and VCAM1 in the Hepa1‐6 cell line and H22 cell line through CLSM and flow cytometry (FCM), respectively. GPC3 and VCAM1 were highly expressed in both cell lines (Figures S7 and S8, Supporting Information). Thus, the Hepa1‐6 cell line and H22 cell line could be applied as HCC cell models for further evaluation. Then, we studied the specific targeting ability of GV‐Lipo/SF/DT by in vitro cellular uptake and competitive inhibition experiments (Figure 1f,g; Figure S9, Supporting Information). The mean fluorescence intensity was quantified, and the intensities of the G‐Lipo/C6 group (*p* < 0.01), V‐Lipo/C6 group (*p* < 0.05) and GV‐Lipo/C6 group (*p* < 0.01) were increased than that of the Lipo/C6 group (Figure 1f). After competitive inhibition with excess free anti‐GPC3 mAb, anti‐VCAM1 mAb, or anti‐GPC3 mAb and anti‐VCAM1 mAb, the mean fluorescence intensity significantly decreased (*p* < 0.01, Figure 1f,g). These results indicated that GPC3 and VCAM1 dual‐targeted liposomes possessed an enhanced specific targeting ability in vitro. The in vivo specific targeting ability of GV‐Lipo/SF/DT was further evaluated by an NIRF imaging study (Figure 1h–j). In that experiment, the near infrared dye IR780 iodide served as the imaging indicator.^[^ ^29^, ^30^ ^]^ Notably, the fluorescence intensity in the GV‐Lipo/IR780 group was much stronger than that in the G‐Lipo/IR780 (*p* < 0.01) and V‐Lipo/IR780 (*p* < 0.01) groups, demonstrating the more specific in vivo dual‐targeting ability of GV‐Lipo/IR780.

Taken together, both the in vitro cellular uptake and in vivo NIRF imaging results suggested that GPC3 and VCAM1 dual‐targeted liposomes exhibited a better tumor‐targeting capability than single‐targeted liposomes, which suggests the potential of increased sensitive targeting for recognition and capture of CTCs.

### 2.2 GV‐Lipo/SF/DT Eliminates CTCs Effectively

**Figure 2:**
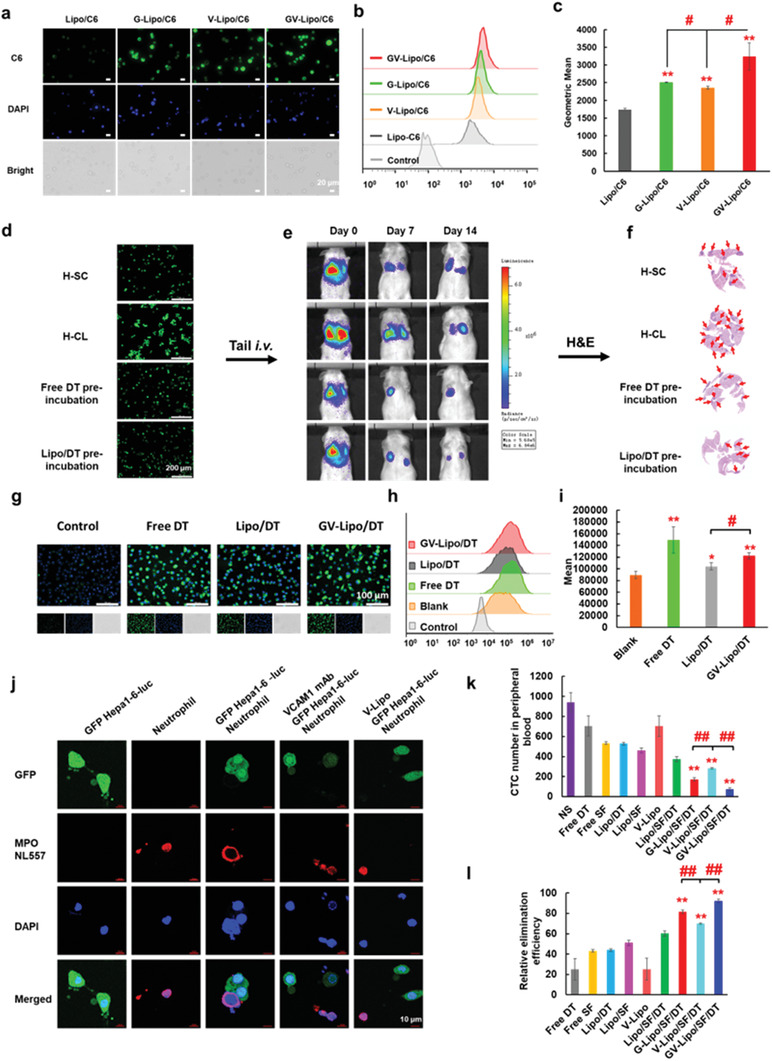
GV‐Lipo/SF/DT eliminates CTCs effectively: GV‐Lipo/SF/DT exhibited specific targeting, which led to recognition and capture of CTCs, dissociation of CTC clusters, and prevention of CTC–neutrophil cluster formation. a–c) GV‐Lipo/SF/DT exhibited specific targeting, resulting in recognition and capture of CTC in vitro. (a) Representative fluorescence images. Scale bar: 20 µm. (b) FCM histogram profiles of fluorescence intensity are shown. (c) The mean fluorescence intensity of cells was determined by FCM. *n* = 3, **p < 0.01 compared to Lipo/C6, ^#^ *p* < 0.05. d–f) DT could dissociate CTC clusters. (d) Representative images of GFP Hepa1‐6‐luc single cells (H‐SL), GFP Hepa1‐6‐luc clusters (H‐CL), free DT‐preincubated H‐CL and Lipo/DT‐preincubated H‐CL are shown. Scale bar: 200 µm. (e) The in vivo bioluminescence of mice was evaluated after i.v. injection of different cells on days 0, 7, and 14 postinjection. (f) H&E images of the lungs on day 14 postinjection are shown. g–i) DT increased the intracellular Ca^2+^ concentration in Hepa1‐6 cells. (g) Representative inverted fluorescence microscopy images of intracellular Ca^2+^ in Hepa1‐6 cells after coincubated with DT, Lipo/DT, and GV‐Lipo/DT (DT concentration: 3 × 10^−6^ m) for 8 h. Scale bar: 100 µm. (h) FCM histogram profiles of fluorescence intensity. (i) Quantification of mean fluorescence intensity by FCM analysis. *n* = 3, *p < 0.05 and **p < 0.01 compared to blank group, ^#^ *p* < 0.05. j) Representative CLSM images show the prevention of the formation of CTC–neutrophil clusters by the anti‐VCAM1 mAb. Scale bar: 10 µm. k–l) GV‐Lipo/SF/DT eliminates associated‐CTCs in vivo. (k) The CTC number in the peripheral blood was determined after different formulations were injected into mice. *n* = 3, **p < 0.01 compared to Lipo/SF/DT, ^##^ *p* < 0.01. l) The relative CTC elimination efficiency of GV‐Lipo/SF/DT in vivo was determined. *n* = 3, **p < 0.01 compared to Lipo/SF/DT, ^##^ *p* < 0.01.

GV‐Lipo/SF/DT exhibited specific targeting allowing recognition and capture of CTCs, dissociation of CTC clusters, and prevention of CTC–neutrophil cluster formation. We systematically evaluated the ability of GV‐Lipo/SF/DT to inhibit the spread of “seeds”. To evaluate the specific targeting ability of GV‐Lipo/SF/DT, Hepa1‐6 cells were used as a CTC model since the metastasis‐promoting glycoproteins, such as VCAM1 and GPC3, were high expressed on Hepa1‐6 cells (Figure S6, Supporting Information). First, the specific targeting of GV‐Lipo/SF/DT to CTCs was evaluated by mimicking the in vivo blood flow environment using a rotary viscometer (shear rate: 188 s^−1^).^[^ ^31^ ^]^ The mean fluorescence intensity of GV‐Lipo/C6‐incubated Hepa1‐6 cells was increased compared to G‐Lipo/C6‐treated Hepa1‐6 cells (*p* < 0.05) and V‐Lipo/C6‐treated Hepa1‐6 cells (*p* < 0.05) after shearing at 37 °C for 0.5 h (**Figure** **2**a–c). These results indicated that GV‐Lipo/SF/DT could recognize and capture CTCs in the bloodstream more specifically than single‐targeted liposomes.

**Figure 3:**
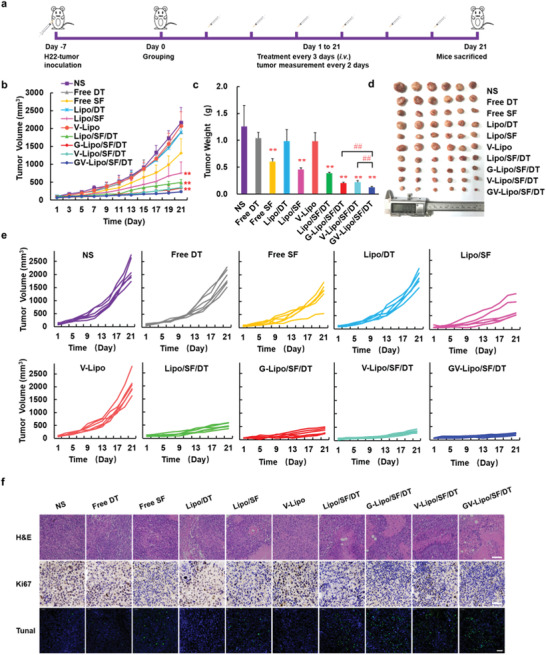
GV‐Lipo/SF/DT showed enhanced antitumor efficacy in H22‐bearing tumor model. a) Schematic illustration of antitumor efficacy in an H22‐bearing tumor model. b) Tumor growth curves during treatment. c) Tumor weight after treatment. d) Photograph of tumors. e) Body weight of H22‐bearing mice treated with NS, free DT, free SF, Lipo/DT, Lipo/SF, V‐Lipo, Lipo/SF/DT, G‐Lipo/SF/DT, V‐Lipo/SF/DT or GV‐Lipo/SF/DT (10 mg kg^−1^ SF, 1.0 mg kg^−1^ DT). **p < 0.01 compared with NS; ^##^ *p* < 0.01 compared with GV‐Lipo/SF/DT. *n* = 6. f) H&E‐, Ki67‐, and TUNEL‐stained images of tumors from different groups. Scale bars for H&E‐, Ki67‐, and TUNEL‐stained images were 100, 50, and 50 µm, respectively.

Next, we evaluated the ability of free DT and Lipo/DT to dissociate CTC clusters of Hepa1‐6 cells in vitro without affecting cellular viability. The preferred concentration of DT to dissociate CTC clusters was screened. Almost no CTC clusters formed when the concentration DT in Lipo/DT was 3 × 10^−6^ m, with no detectable reduction in cell viability (>70% viability, Figure S10, Supporting Information).^[^ ^11^ ^]^ Thus, we chose 3 × 10^−6^ m DT as the concentration for dissociating CTC clusters in vitro. To test whether DT could dissociate CTC clusters in vivo, we carried out an in vivo metastasis model study with four different groups. GFP Hepa1‐6‐luc single cells (H‐SL), GFP Hepa1‐6‐luc clusters (H‐CL), or H‐CL preincubated with free DT or Lipo/DT (**Figure** **3**d) were intravenously (i.v.) injected into mice, and pulmonary metastasis was observed by bioluminescence imaging. On day 14 after cell injection, the bioluminescence signal in the lungs of mice in the H‐CL group was stronger than that in the other three groups (Figure 2e). Additionally, there were more metastatic tumor nodes in the H‐CL group than in the other groups, as determined by hematoxylin and eosin (H&E) staining (Figure 2f). Taken together, these results indicated that CTC clusters increased tumor metastasis compared with single CTCs, while DT could dissociate CTC clusters, leading to a reduction in HCC pulmonary metastasis. Na^+^/K^+^ ATPase inhibitors, such as DT, can increase the intracellular Ca^2+^ concentration, thereby preventing cancer cells from forming cell–cell junctions and thus dissociating CTC clusters.^[^ ^32^, ^33^ ^]^ Therefore, we assessed the intracellular Ca^2+^ concentration in Hepa1‐6 cells after treatment with DT, Lipo/DT or GV‐Lipo/DT (DT concentration: 3 × 10^−6^ m) for 8 h. We found that the intracellular Ca^2+^ concentration increased significantly upon treatment with DT (*p* < 0.01), Lipo/DT (*p* < 0.05) or GV‐Lipo/DT (*p* < 0.01) compared with no treatment (Figure 2g–i). The results indicate that DT dissociates CTC clusters by increasing the intracellular Ca^2+^ concentration.

The ability of the anti‐VCAM1 mAb to prevent the formation of CTC–neutrophil clusters was evaluated using neutrophils cocultured with GFP Hepa1‐6‐luc cells. CTC–neutrophil clusters formed when neutrophils and GFP Hepa1‐6‐luc cells were cocultured in 1640 medium overnight (Figure 2j). By contrast, in the context of preincubation with the anti‐VCAM1 mAb or V‐lipo, the number of formed CTC–neutrophil clusters was obviously decreased, which indicated that the competitive binding of the anti‐VCAM1 mAb to VCAM1 could prevent the formation of CTC–neutrophil clusters.

Furthermore, the final effect, namely, GV‐Lipo/SF/DT inhibiting the spread of “seeds” in the blood circulatory system in vivo, was examined. GFP Hepa1‐6‐luc cells were intravenously injected into mice as a CTC simulant, followed by intravenous administration of different formulations. At 24 h postadministration, the CTCs in the blood were separated and purified and quantified by FCM. As shown in Figure 2k–l, the CTC elimination efficiency of GV‐Lipo/SF/DT (92.24% ± 1.60%) was significantly increased compared to G‐Lipo/SF/DT (81.72% ± 1.74%, *p* < 0.01) or V‐Lipo/SF/DT (70.07% ± 0.75%, *p* < 0.01), which confirmed that dual‐targeted liposomes could recognize and capture CTCs more specifically in vivo, thus inhibiting CTCs more efficiently.

Overall, GV‐Lipo/SF/DT effectively inhibited the spread of “seeds”. When GV‐Lipo/SF/DT circulated in the bloodstream, the anti‐GPC3 mAb and anti‐VCAM1 mAb could specifically recognize and capture CTCs and CTC clusters, and DT could dissociate CTC clusters by increasing the intracellular Ca^2+^ concentration. The competitive binding of the anti‐VCAM1 mAb to VCAM1, which is highly expressed on the surface of CTCs, could prevent the formation of CTC–neutrophil clusters. SF eliminated associated‐CTCs after cellular uptake.

### 2.3 GV‐Lipo/SF/DT Enhanced Antitumor Efficacy in H22‐Bearing Tumor Model

The in vivo antitumor efficacy of GV‐Lipo/SF/DT was evaluated in an H22‐bearing tumor model (Figure 3). As presented in Figure 3b,e, the tumor volume of the saline (NS) group exhibited a quick increase (≈2000 mm^3^ at day 21). Compared with that of the NS group, the tumor volumes of the free DT, Lipo/DT, and V‐lipo groups were not significantly different. Notably, the GV‐Lipo/SF/DT group exhibited the strongest tumor inhibition compared with the Lipo/SF/DT (*p* < 0.01), G‐Lipo/SF/DT (*p* < 0.01) and V‐Lipo/SF/DT (*p* < 0.01) groups. Furthermore, the tumor weight in the GV‐Lipo/SF/DT group was the lowest, with an inhibition rate of 90.28% (Figure 3c; Table S3, Supporting Information). Tumor photographs were consistent with the tumor volume and tumor weight results (Figure 3d). Interestingly, we found that Lipo/SF/DT (tumor inhibition rate: 69.10%) produced better inhibition of tumor volume than Lipo/SF (tumor inhibition rate: 63.68%), while Lipo/DT was not effective in inhibiting tumor growth than NS group (*p* < 0.05). We hypothesized that SF and DT at experimental concentrations might have combined antitumor effects. Thus, possible reasons for the antitumor effect of DT and SF combination were further explored. Studies have shown that DT combined with SF could inhibit HCC by cell cycle arrest.^[^ ^34^ ^]^ The effects of SF and DT on the cell cycle in vitro were examined. Cell cycle arrest was induced in the G1 phase after treatment with Lipo/SF/DT (Figure S11, Supporting Information), which indicated that SF and DT had a certain combined effect producing cell cycle arrest. As studied above, DT could dissociate CTC clusters to single CTC. We hypothesized that DT might be able to dissociate tumor cells in tumor tissue, promote deep penetration of SF, and improve the therapeutic effect. A large number of studies using traditional strategy, such as modulation of tumor microenvironments and optimization of nanoparticles, to promote deep tumor penetration and showed some effect.^[^ ^35^ ^]^ To evaluate whether DT could enhance the penetration ability of liposomes, the deep penetration ability of GV‐Lipo/C6 was studied in Hepa1‐6 tumor spheres. The tumor penetration ability of GV‐Lipo/SF/DT was enhanced in Hepa1‐6 tumor spheres after preincubated with free DT, Lipo/DT, and GV‐Lipo/DT (DT concentration: 3 × 10^−6^ m), which may be because DT induced the cancer cells to lose cell–cell junctions to some extent, and thus conducive to the deep penetration of liposomes (Figure S12, Supporting Information). Taken together, DT at experimental concentrations promoted the antitumor effect of SF, which due to DT had a certain combined effect with SF by producing cell cycle arrest and enhanced the deep penetration of SF by lose cell–cell junctions to some extent.

As shown in Figure 3f, nuclear shrinkage occurred in free SF group, Lipo/SF group, Lipo/SF/DT group, G‐Lipo/SF/DT group, V‐Lipo/SF/DT group, and GV‐Lipo/SF/DT group in H&E staining, which indicated the necrosis and apoptosis of the tumor cells. Similar results were shown in the TUNEL assay. The green fluorescence‐labeled cells were more in Lipo/SF/DT group, G‐Lipo/SF/DT group, V‐Lipo/SF/DT group, and GV‐Lipo/SF/DT group. On the contrary, fewer Ki67‐positive cells were stained in Lipo/SF/DT group, G‐Lipo/SF/DT group, V‐Lipo/SF/DT group, and GV‐Lipo/SF/DT group, which exhibited that proliferation was inhibited after treatment. The GV‐Lipo/SF/DT group exhibited more shrinkage of the nucleus in H&E‐stained images, fewer Ki67‐positive cells and more green fluorescence‐labeled cells by TUNEL staining, indicating that GV‐Lipo/SF/DT could effectively inhibit the proliferation and enhance the apoptosis of tumor cells. In summary, GV‐Lipo/SF/DT enhanced antitumor efficacy in vivo through dual targeting and a multifunctional combination in an H22‐bearing tumor model.

A preliminary safety evaluation of each formulation was carried out; a hemolysis test, mouse body weight measurements, and H&E staining of main organs were performed. No hemolysis was observed even at the highest concentration of GV‐Lipo/SF/DT (Figure S13a, Supporting Information). The hemolytic ratio of GV‐Lipo/SF/DT was negligible when the concentration of SF ranged from 13 to 65 µg mL^−1^ (<5%) (Figure S13b, Supporting Information). There were no significant changes in the body weights of mice in different groups during treatment (Figure S14, Supporting Information). H&E images of main organs showed no visual pathological changes in cellular morphology in any of the treatment groups (Figure S15, Supporting Information). Thus, GV‐Lipo/SF/DT exhibited good biocompatibility without gross toxicity.

### 2.4 GV‐Lipo/SF/DT Exhibited Superior Antitumor Efficacy in Orthotopic HCC Models In Vivo

**Figure 4:**
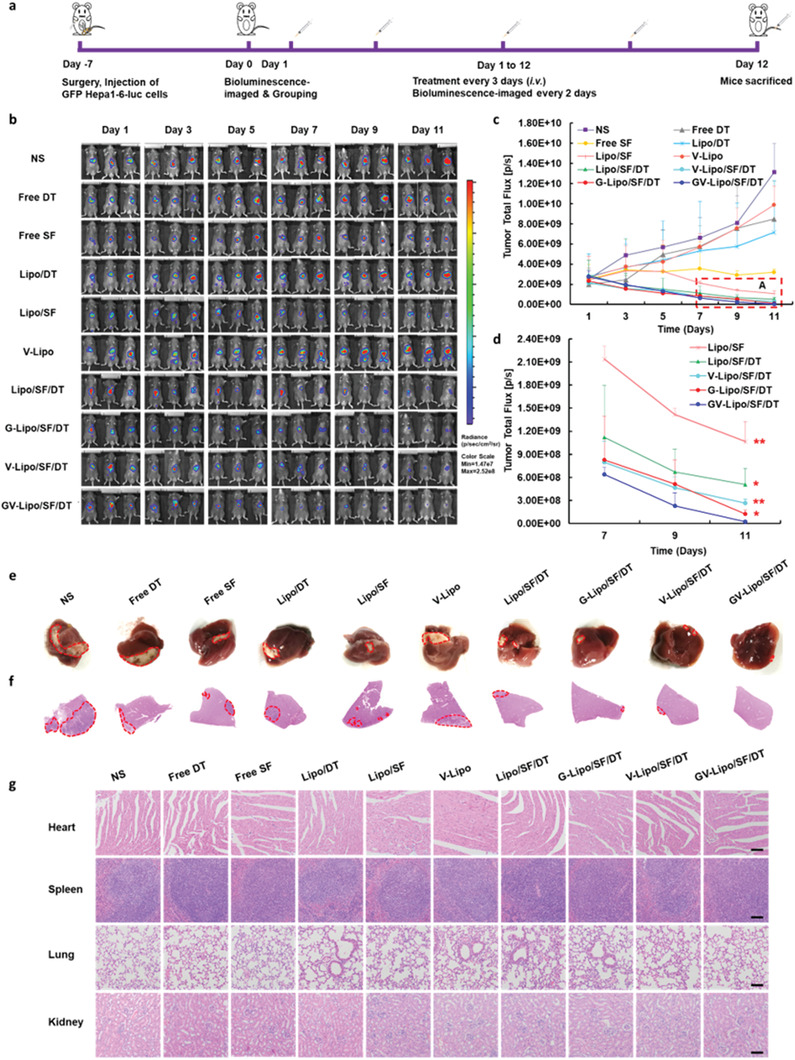
GV‐Lipo/SF/DT exhibited superior antitumor efficacy in orthotopic HCC model mice in vivo. a) Schematic illustration of antitumor efficacy in male GFP Hepa1‐6‐luc tumor bearing C57BL/6 orthotopic HCC model mice. b) In vivo bioluminescence images of mice during treatment. c) Bioluminescence intensity changes in tumor tissues based on bioluminescence imaging of mice. d) An enlarged view of area A in (c). **p < 0.01 compared with GV‐Lipo/SF/DT. *n* = 3. e) Representative photographs of livers from orthotopic HCC mice in each group after treatment. f) H&E‐stained left lobe of the liver from orthotopic HCC mice. Red dotted circles indicate the tumor area. g) Representative H&E staining of heart, spleen, lung, and kidneys of orthotopic HCC mice. Scale bars: 100 µm.

The antitumor efficacy of GV‐Lipo/SF/DT was further evaluated in orthotopic GFP Hepa1‐6‐luc tumor‐bearing mice (**Figure** **4**; Figure S16, Supporting Information),^[^ ^36^ ^]^ because the orthotopic tumor model has the most clinically significance mimicking human hepatomas in terms of the tumor microenvironment, morphology, and metastatic potential.^[^ ^37^ ^]^ As shown in Figure 4b–d, the bioluminescence intensity in the NS group showed a 5.18‐fold enhancement (from 2.53 × 10^9^ p s^−1^ cm^−2^ sr^−1^ on day 1 to 1.16 × 10^10^ p s^−1^ cm^−2^ sr^−1^ on day 11). The bioluminescence intensities in the free DT, Lipo/DT, and V‐lipo groups were enhanced on the last day and showed no significant differences in comparison with that in the NS group, which indicated that free DT, Lipo/DT, and V‐lipo could not inhibit tumor growth effectively. However, the bioluminescence intensity in the free SF group did not significantly increase from day 1 to day 11, indicating that free SF could inhibit tumor growth. Compared to that in the NS group, the bioluminescence intensities in all other groups were significantly decreased on day 11 (*p* < 0.01). Notably, the bioluminescence intensity in the GV‐Lipo/SF/DT group was the lowest compared with that in the Lipo/SF (*p* < 0.01), Lipo/SF/DT (*p* < 0.05), G‐Lipo/SF/DT (*p* < 0.05), and V‐Lipo/SF/DT (*p* < 0.01) groups, which also indicated that GPC3 and VCAM1 dual targeting of GV‐Lipo/SF/DT could enhance the antitumor effects compared with the single targeting of G‐Lipo/SF/DT and V‐Lipo/SF/DT. These results suggested that GV‐Lipo/SF/DT exhibited superior antitumor efficiency in orthotopic HCC model mice. Body weight showed no significant change after various treatments (Figure S17, Supporting Information). Representative photographs of livers from orthotopic HCC mice after treatment were shown in Figure 4e. The tumor tissues in the livers were marked with red dotted circles, and the tumor volume was the smallest in the GV‐Lipo/SF/DT group. H&E staining of the liver also presented the same trend, with the smallest tumor tissues in the GV‐Lipo/SF/DT group (Figure 4f). Additionally, images of H&E‐stained major organs showed no visual signs of systemic toxicity (Figure 4g). In brief, GV‐Lipo/SF/DT exhibited superior antitumor effects and desirable safety in an orthotopic HCC mouse model.

### 2.5 GV‐Lipo/SF/DT Effectively Prevents the Recurrence and Metastasis of HCC

**Figure 5:**
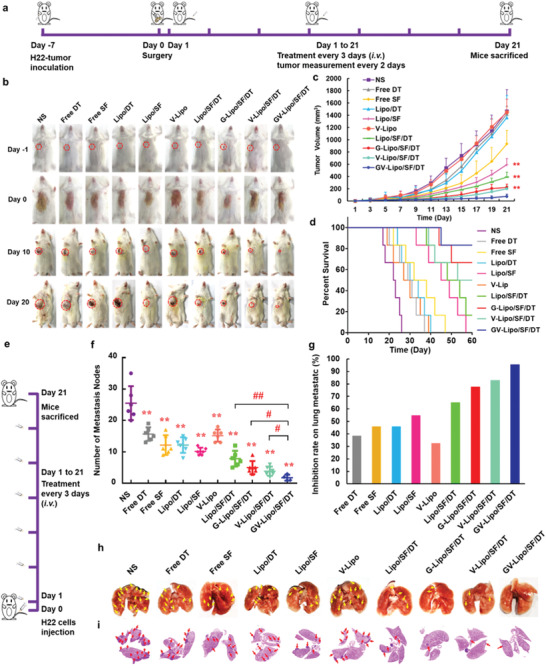
GV‐Lipo/SF/DT effectively prevents HCC recurrence and metastasis. a–d) GV‐Lipo/SF/DT significantly reduced tumor size and enhanced overall survival in a postsurgical H22 tumor‐bearing KM mouse model. (a) Schematic illustration of the antitumor recurrence efficacy experiment. (b) Photographs of postsurgery mice during treatment. (c) Tumor growth curves of different groups postsurgery. (d) Survival curves of different groups postsurgery. *n* = 6, **p < 0.01 compared to GV‐Lipo/SF/DT. e–h) GV‐Lipo/SF/DT effectively inhibited tumor pulmonary metastasis in a KM mouse metastasis model. The KM mouse metastasis model was established via intravenous injection of H22 cells (8 × 10^6^ H22 cells per mouse), *n* = 6. (e) Schematic illustration of the pulmonary metastasis inhibition study. (f) The number of pulmonary metastasis nodes in different groups. **p < 0.01 compared to NS. ^#^ *p* < 0.05, ^##^ *p* < 0.01. (g) Rates of lung metastasis inhibition in different groups. (h) Representative photographs of the lungs. The yellow arrow indicates metastatic nodes. i) Representative H&E images of lungs from metastasis model mice after treatment. The red arrow indicates metastatic nodes.

The ability of GV‐Lipo/SF/DT to inhibit tumor recurrence after surgical resection was evaluated in a postsurgical H22 tumor‐bearing KM mouse model (**Figure** **5**a–d). Compared with free SF, different liposomal formulations presented better inhibitory effects on tumor recurrence and produced a higher mouse survival rate (Figure 5b–d; Figure S18, Supporting Information). Notably, GV‐Lipo/SF/DT exhibited superior inhibitory effects and enhanced overall survival compared with single‐targeted G‐Lipo/SF/DT and V‐Lipo/SF/DT, which indicated that dual‐targeting liposomes exhibited a more sensitive tumor‐targeting ability than single‐targeted liposomes. No significant changes in body weight were observed in the groups during treatment (Figure S19, Supporting Information).

The inhibitory effect of GV‐Lipo/SF/DT on pulmonary metastasis was evaluated in a mouse metastasis model via intravenous injection of H22 cells (8 × 10^6^ H22 cells per mouse) (Figure 5e–i). The number of pulmonary metastasis nodes was decreased in the free DT (*p* < 0.01), Lipo/DT (*p* < 0.01), and V‐Lipo (*p* < 0.01) groups compared with the NS group, which indicated that DT and the anti‐VCAM1 mAb could prevent metastasis to a certain extent. Additionally, the number of pulmonary metastasis nodes observed after treatment with different formulations (free SF (*p* < 0.01), Lipo/SF (*p* < 0.01), Lipo/SF/DT (*p* < 0.01), G‐Lipo/SF/DT (*p* < 0.01), V‐Lipo/SF/DT (*p* < 0.01), and GV‐Lipo/SF/DT (*p* < 0.01)) was significantly decreased in these groups compared with the NS group. Significantly, the number of pulmonary metastasis nodes after GV‐Lipo/SF/DT treatment was less than that after treatment with Lipo/SF/DT (*p* < 0.01), G‐Lipo/SF/DT (*p* < 0.05) or V‐Lipo/SF/DT (*p* < 0.05) (Figure 5f), with an inhibition rate of up to 95.42% (Figure 5g). Representative photographs of the lungs and H&E images from different groups showed similar results (Figure 5h,i), with the fewest pulmonary metastasis nodes observed in the GV‐Lipo/SF/DT group. It is difficult to observe how anti‐VCAM1 mAb prevents the formation of CTC–neutrophil clusters during blood circulation in vivo. Thus, neutrophils were stained at pulmonary metastasis nodules after treatment with different formulations. Compared with NS group, the number of neutrophils (red fluorescence) was reduced in V‐Lipo group, V‐Lipo/SF/DT group, and GV‐Lipo/SF/DT in pulmonary metastasis nodules (Figure S20, Supporting Information), the results indicated that preventing CTC–neutrophil cluster formation with the anti‐VCAM1 mAb might reduce the number of neutrophils at the metastatic nodules.

GV‐Lipo/SF/DT could effectively inhibit the recurrence and metastasis of HCC, clarifying that dual targeting of GPC3 and VCAM1 could enhance the recognition of and uptake by tumor cells, thus killing primary tumor cells by SF, DT could dissociate CTC clusters and the anti‐VCAM1 mAb could prevent the formation of CTC–neutrophil clusters, all of which inhibited tumor metastasis together.

## 3 Conclusion

In summary, primary tumor and associated‐CTCs multipoint costriking nanodevice (GV‐Lipo/SF/DT) enhanced metastasis inhibition and therapeutic effect on HCC. GV‐Lipo/SF/DT inhibited the recurrence and metastasis effectively (inhibition rate 95.42%) by specific targeting of associated‐CTCs, dissociating CTC clusters, preventing CTC–neutrophil cluster formation and eliminating CTCs. Importantly, GV‐Lipo/SF/DT showed superior antitumor effects on the H22‐bearing tumor model (inhibition rate 90.28%) and orthotopic HCC models (inhibition rate 99.81% calculated by fluorescence intensity) in vivo with remarkable tumor suppression. The FDA approved the liposome Vyxeos (coloading of daunorubicin and cytarabine for injection) to treat acute myeloid leukaemia in 2017,^[^ ^38^ ^]^ which indicated that GV‐Lipo/SF/DT liposomes with SF and DT coloaded might have great translational potentials. Overall, the “roots and seeds” multipoint costriking strategy has opened a new possibility for omnidirectional inhibition of tumor metastasis, which could be translated into a new treatment concept for various cancers in the clinic.

CTCs are closely related to cancer metastasis and recurrence. Along with the development of CTC isolation technologies and in‐depth study of CTCs, CTCs have begun to be considered an important therapeutic target, and adopting therapeutic strategies to eliminate CTCs would be a new and feasible way to inhibit cancer metastasis or recurrence. At present, Na^+^/K^+^ ATPase inhibitors digoxin has been used to dissociate CTC clusters in phase I clinical study (NCT03928210). However, associated‐CTCs, such as CTC clusters, CTC–neutrophil clusters, and other CTC forms, promote metastasis through various immune escape mechanisms. For example, apart from single CTCs, CTC clusters, and CTC–neutrophil clusters, platelets promote tumor metastasis by aggregating with CTCs, thus helping CTCs avoid immune attack and metastasis.^[^ ^39^ ^]^ In order to target associated‐CTCs, additional studies are needed to explain how CTCs escape from immune surveillance. More further research on the relationships between associated‐CTC formation and immune escape, and targeting associated‐CTCs strategies should be carried out. In consequence, targeting associated‐CTCs strategy is a promising avenue to inhibit tumor metastasis and has great clinical application prospects.

## Conflict of Interest

The authors declare no conflict of interest.

## Supporting information

Supporting Information

## Data Availability

The data that support the findings of this study are available from the corresponding author upon reasonable request.
