# Supplementary material for: Multipoint Costriking Nanodevice Eliminates Primary Tumor Cells and Associated‐Circulating Tumor Cells for Enhancing Metastasis Inhibition and Therapeutic Effect on HCC
Source: Adv Sci (Weinh). 2022 Jan 14;9(9):2101472. doi: 10.1002/advs.202101472 (PMC8948568; doi:10.1002/advs.202101472)
Supplement: Supplementary file 1 — Supporting Information [file ADVS-9-2101472-s001.pdf]

ADVANCED SCIENCE
Open Access

Supporting Information

for *Adv. Sci.*, DOI 10.1002/advs.202101472

Multipoint Costriking Nanodevice Eliminates Primary Tumor Cells and Associated-Circulating Tumor Cells for Enhancing Metastasis Inhibition and Therapeutic Effect on HCC

Weiwei Mu, Qihui Chu, Huizhen Yang, Li Guan, Shunli Fu, Tong Gao, Xiao Sang, Zipeng Zhang, Shuang Liang, Yongjun Liu and Na Zhang**

WILEY-VCH

ADVANCED SCIENCE
Open Access

Supporting Information

for *Adv. Sci.*, DOI: 10.1002/advs.202101472

Multipoint Co-striking Nanodevice Eliminates Primary Tumor Cells and Associated-Circulating Tumor Cells for Enhancing Metastasis Inhibition and Therapeutic Effect on HCC

*Weiwei Mu, Qihui Chu, Huizhen Yang, Li Guan, Shunli Fu, Tong Gao, Xiao Sang, Zipeng Zhang, Shuang Liang, Yongjun Liu *, Na Zhang **

Supporting Information

Multipoint Co-striking Nanodevice Eliminates Primary Tumor Cells and Associated-Circulating Tumor Cells for Enhancing Metastasis Inhibition and Therapeutic Effect on HCC

*Weiwei Mu, Qihui Chu, Huizhen Yang, Li Guan, Shunli Fu, Tong Gao, Xiao Sang, Zipeng Zhang, Shuang Liang, Yongjun Liu *, Na Zhang **

Part A. Experimental Section

Cells and materials. The Hepa1-6 and GFP Hepa1-6-luc cell lines were obtained from the Institute of Immunopharmaceutical Science of Shandong University (Jinan, PRC). H22 cells were cultured from murine H22 ascites sarcoma. Hepa1-6 cells were cultured in high-glucose Dulbecco's modified Eagle's medium (DMEM, Gibco) supplemented with 10% fetal bovine serum (FBS). GFP Hepa1-6-luc cells were cultured in DMEM (Gibco) supplemented with 10% FBS and 0.1% puromycin.

SF was purchased from Shanghai Biochempartner Co., Ltd. DT and cholesterol (Chol) were purchased from Sigma-Aldrich. Egg phosphatidylcholine (EPC) was obtained from AVT Pharmaceutical Technology Co., Ltd. A FITC-labelled anti-GPC3 mAb was purchased from Wuhan Biorbyt Biotechnology Co., Ltd. (no. orb360789). A FITC-labelled anti-VCAM1 antibody was purchased from Abcam (no. ab24853).

Synthesis of functional materials. A DSPE-PEG$_{2000}$-anti-GPC3 mAb and DSPE-PEG$_{2000}$-anti-VCAM1 mAb were synthesized by Michael's addition reaction of maleimide with a sulfhydryl group. Briefly, an anti-GPC3 mAb-SH was obtained by reaction of the anti-GPC3 mAb with Traut’s reagent. The anti-GPC3 mAb was diluted in PBS (pH 7.0), and then Traut’s reagent (10 Eq) was added. The mixture was stirred for 60 min at room temperature. Excess Traut’s reagent was removed with a Zeba desalt spin column (Thermo Scientific-Pierce). The anti-GPC3 mAb-SH was evaluated using Ellman’s reagent. After purification, the anti-GPC3 mAb-SH (1.0 Eq) and DSPE-PEG$_{2000}$-maleimide (1.2 Eq) were

dissolved in PBS (pH 7.4) and stirred for 8 h at room temperature. The product was purified by dialysis (MWCO 5000 Da, Millipore) for 2 days. After lyophilization, the product was verified by ^{1}H NMR (Avance™ DPX-300; Bruker BioSpin GmbH, Rheinstetten). The DSPE-PEG$_{2000}$-anti-VCAM1 mAb was synthesized by the same method except that the anti-GPC3 mAb was replaced with the anti-VCAM1 mAb.

Expression of GPC3 and VCAM1. The expression of GPC3 and VCAM1 in the Hepa1-6 cell line and H22 cell line was analyzed. Briefly, Hepa1-6 cells were seeded in a confocal dish (1×10^{5} cells/well) and incubated overnight. Then, fresh PBS (pH 7.4) containing a FITC-labelled anti-GPC3 mAb (200 ng/mL) was added and incubated for 0.5 h on ice. After the incubation, the cells were fixed in 4% paraformaldehyde and then stained with 4′,6-diamidino-2-phenylindole (DAPI) for 10 min at room temperature. Finally, the dish was examined under CLSM (LSM 780, Carl Zeiss). To quantify the expression of GPC3, Hepa1-6 cells were digested after incubation with the FITC-labelled anti-GPC3 mAb, centrifuged, resuspended in cold PBS (pH 7.4) and detected by a flow cytometer (BD FACSCelesta). To verify the expression of GPC3 in H22 cells, H22 cells were added to a 2-mL centrifuge tube at a density of 1×10^{5} and analyzed using the method described above. The expression of VCAM1 in the Hepa1-6 cell line and H22 cell line was analyzed using the same method, except the FITC-labelled anti-GPC3 mAb was replaced with a FITC-labelled anti-VCAM1 mAb.

Preparation and characterization of GV-Lipo/SF/DT. GV-Lipo/SF/DT was prepared by the film dispersion method. Briefly, EPC (80 mg), Chol (10 mg), SF (5.2 mg), and DT (16 μg) were dissolved in 4 mL ethanol solution. The lipid film was formed by rotary evaporation at 40 °C, and the DSPE-PEG$_{2000}$-anti-GPC3 mAb and DSPE-PEG$_{2000}$-anti-VCAM1 mAb (3% mol ratio relative to total phospholipids for each) were added during hydration. GV-Lipo/SF/DT was extruded through 0.45-μm and 0.22-μm membrane filters and subsequently using a LiposoFast™-Basic extruder (0.20 μm and 0.10 μm) (Avestin, Ottawa,

ON) five times. The particle size, polydispersity index (PDI), and zeta potential of GV-Lipo/SF/DT were determined with a Zetasizer Nano ZS90 (Malvern, Worcestershire). The morphology of GV-Lipo/SF/DT was evaluated by transmission electron microscopy (TEM). The EE% and drug loading (DL%) of SF and DT were calculated using the following equations:

$$DL\% = (W_{\text{loaded drug}}/W_{\text{liposomes}}) \times 100\% \quad (1)$$

$$EE\% = (W_{\text{loaded drug}}/W_{\text{total drug}}) \times 100\% \quad (2)$$

where $W_{\text{loaded drug}}$ represents the amounts of loaded drug in GV-Lipo/SF/DT and $W_{\text{total drug}}$ and $W_{\text{liposomes}}$ represent the amounts of added drug and liposomes, respectively. All samples were evaluated in triplicate.

To verify that the targeted antibodies were modified on the surface of the liposomes, the anti-GPC3 mAb was labeled with FITC, and the anti-VCAM1 mAb was labeled with RhB. Then, fluorescent dye-labeled GV-Lipo/SF/DT was imaged by CLSM. The amount of modification by the targeting antibodies was analyzed with BCA Protein Assay Reagent. The modification rates of the anti-GPC3 mAb or the anti-VCAM1 mAb on liposomes were calculated using the following equations:

$$\text{Modification rates (\%)} = (W_{\text{modified mAb}} / W_{\text{total mAb}}) \times 100\% \quad (3)$$

where $W_{\text{modified mAb}}$ represents the amounts of anti-GPC3 mAb or anti-VCAM1 mAb modified on the surface of GV-Lipo/SF/DT and $W_{\text{total drug}}$ represent the amounts of added anti-GPC3 mAb or anti-VCAM1 mAb when GV-Lipo/SF/DT prepared.

Stability of GV-Lipo/SF/DT in different media. The storage stability of GV-Lipo/SF/DT was evaluated for a total of 30 days. Samples were prepared under aseptic conditions and stored at 4 °C. Particle size was determined every 3 days. Preliminary plasma (20%, v/v) stability was also investigated. The particle size of GV-Lipo/SF/DT was measured after incubation in 20% plasma for 0 h, 2 h, 4 h, 8 h, 12 h, 24 h, 48 h, and 72 h at 37 °C.

Cumulative release of SF and DT. The release profiles of SF and DT from GV-Lipo/SF/DT were evaluated using 0.1 M PBS (pH 7.4, containing 1% Tween-80) as the release medium. Briefly, free SF, free DT and GV-Lipo/SF/DT were suspended in PBS (pH 7.4, containing 1% Tween-80). Each dialysis bag contained 1.5 mL sample (final concentrations: 20 μg/mL SF and 15 μg/mL DT) and 15 mL release medium and shaking in a constant temperature water bath at 37 ℃. At different pre-set time points, the solution was harvested and replaced. The concentrations of SF and DT were determined by high-performance liquid chromatography (HPLC). Each group was evaluated in triplicate.

In vitro cytotoxicity assay. Hepa1-6 cells (5000 cells/well) were exposed to blank liposomes (blank Lipo), free SF, free DT, Lipo/SF, Lipo/DT, Lipo/SF/DT or GV-Lipo/SF/DT for 48 h. After treatment, 20 μL 3-(4,5-dimethylthiazol-2-yl)-2,5-diphenyltetrazolium bromide (MTT, 5 mg/mL) was added and incubated at 37 °C for 4 h. Then, the medium was removed, and 150 μL DMSO was added. The untreated cells group was set as the control group and PBS group was set as blank group. The cell viability in each group was measured using a microplate reader (Model 680; Bio-Rad, CA). All experiments were carried out in triplicate. Relative cell viability (%) was calculated using the following equation:

$$\text{Relative cell viability (\%)} = \left(\frac{A_{control} - A_{sample}}{A_{control} - A_{blank}}\right) \times 100 \ \ (\%) \qquad (4)$$

where A_{sample} represents the absorbance value of different formulation, $A_{control}$ represents the absorbance value of control group, and A_{blank} represents the absorbance value of blank group.

In vitro targeting assay evaluating GV-Lipo/SF/DT. The targeting ability of the formulation was evaluated in an *in vitro* cellular uptake experiment. C6 was used as the fluorescence indicator in the cellular uptake experiment. Hepa1-6 cells were seeded overnight in a 12-well plate (1×10^5 cells/well). After discarding the medium, fresh DMEM containing free C6, C6-loaded liposomes (C6-Lipo), anti-GPC3 mAb-modified C6-loaded liposomes (G-Lipo/C6), anti-VCAM1 mAb-modified C6-loaded liposomes (V-Lipo/C6), or anti-GPC3 mAb and

anti-VCAM1 mAb dual-modified C6-loaded liposomes (GV-Lipo/C6) (C6 concentration: 200 ng/mL) was added and further incubated for 0.5 h. Then, the cells were washed twice with PBS (pH 7.4) and stained with DAPI for 10 min. Finally, the cells were imaged using an imaging reader (BioTek Cytation 1). To quantify the targeting abilities of the formulations, after treatment with free C6, C6-Lipo, G-Lipo/C6, V-Lipo/C6 or GV-Lipo/C6, the Hepa1-6 cells were digested, centrifuged, re-suspended and detected by FCM. Furthermore, a competitive inhibition experiment was used to evaluate the targeting properties of GV-Lipo/C6. After pre-incubation with excess free anti-GPC3 mAb, anti-VCAM1 mAb, or anti-GPC3 mAb + anti-VCAM1 mAb, GPC3 and/or VCAM1 were saturated. Following the addition of G-Lipo/C6, V-Lipo/C6 or GV-Lipo/C6 into the wells and incubation for 0.5 h, the cells were imaged using an imaging reader and FCM (BD FACSCelesta).

Specific targeting capture of GV-Lipo/SF/DT to CTCs in vitro. To mimic the blood flow environment *in vivo*, a rotary viscometer (Brookfield Engineering Laboratories, Inc., Middleboro) was selected to provide a physical shear force for CTCs. Hepa1-6 cells were digested and washed with PBS, and Hepa1-6 cells (1×10^6) were suspended in 1 mL DMEM containing C6-Lipo, G-Lipo/C6, V-Lipo/C6 or GV-Lipo/C6 (C6 concentration: 200 ng/mL). Then, the suspended cells were incubated under the action of the rotary viscometer (shear rate: 188 s^{-1}) for 0.5 h at 37 °C. After the incubation, the suspended cells were centrifuged to remove the medium and stained with DAPI for 10 min. Finally, the cells were imaged using an imaging reader and quantified by FCM.

In vitro CTC cluster dissociation assay. To investigate the CTC cluster dissociation ability of DT, free DT and Lipo/DT were resuspended in Hepa1-6 CTC medium (5000 cells/well) at different concentrations (0, 0.5, 1, 1.5, 2, 3, 4, and 5 μM) and incubated for 48 h in 96-well plates. After the incubation, cell morphology was examined with an imaging reader (BioTek Cytation 1). Cytotoxicity was further studied; 20 μL of 5 mg/mL MTT was added and

incubated at 37 °C for 4 h. Then, DMSO was added, and cell viability was measured and calculated as described in the "*In vitro cytotoxicity assay*" subsection.

Intracellular Ca^{2+} concentration determination after treatment with DT. To study whether DT influences the intracellular Ca^{2+} concentration, the intracellular Ca^{2+} concentration in Hepa1-6 cells was measured by BBcellProbe®F03 (Shanghai BestBio Biotechnology Co., Ltd.). Briefly, Hepa1-6 cells were incubated overnight in a 12-well plate at a density of 1×10^5 cells/well. After discarding the medium, fresh medium containing free DT, Lipo/DT or GV-Lipo/DT (DT concentration: 3 μM) was added and further incubated for 8 h. Then, the cells were washed three times with cold PBS (pH 7.4), and BBcellProbe®F03 (200 μL/well, 1:500 dilution, excitation wavelength: 488-495 nm, emission wavelength: 516 nm) was added and incubated at 37 °C for 30 min. After the incubation, the cells were stained with DAPI for 10 min. Finally, the cells were imaged using an imaging reader (BioTek Cytation 1). To quantify the intracellular Ca^{2+} concentration, after treatment with DT, Lipo/DT or GV-Lipo/DT (DT concentration: 3 μM), Hepa1-6 cells were digested, centrifuged, re-suspended and detected by FCM (BD FACSCelesta).

Isolation of neutrophils from mouse peripheral blood. Neutrophils were obtained from the whole blood of C57BL/6 mice with a Percoll gradient centrifugation method using a peripheral blood neutrophil isolation kit (Solarbio, PRC). Blood samples were collected in heparin tubes on a sterile test bench and diluted with PBS. Reagent A (4 mL), reagent C (2 mL) and whole blood diluent (2 mL) were added to 15-mL centrifuge tubes and centrifuged at 800 g for 30 min at room temperature. After centrifugation, the neutrophil layer was carefully collected and washed twice with PBS (250 g, 10 min) to obtain high-purity neutrophils. Lipopolysaccharide (LPS, 100 μg/mL) was used to activate neutrophils in serum-free 1640 medium for 6 h.

Prevention of CTC-neutrophil cluster formation in vitro. Neutrophils co-cultured with GFP Hepa1-6-luc cells were used to evaluate the ability of the anti-VCAM1 mAb to prevent the

formation of CTC-neutrophil clusters. GFP Hepa1-6-luc cells (4×10^5) and neutrophils (8×10^5) were seeded in a confocal dish and co-incubated overnight. The following groups were included: the GFP Hepa1-6-luc cell + neutrophil co-incubation group, anti-VCAM1 mAb + GFP Hepa1-6-luc cell + neutrophil co-incubation group and V-lipo + GFP Hepa1-6-luc cell + neutrophil co-incubation group. After the incubation, the cells were washed with PBS (pH 7.4), and the neutrophils were cultured with anti-myeloperoxidase (MPO) antibody (15 μg/mL, Catalogue number: AF3667, R&D Systems) for 3 h. Then, the neutrophils were stained using a NorthernLights 557-conjugated anti-goat IgG secondary antibody (Catalogue number: NL001, R&D Systems) for 0.5 h. The GFP Hepa1-6-luc cells and neutrophils were stained with DAPI for 10 min. Finally, the cells were imaged using CLSM.

Cell cycle assay. The effects of SF and DT on the cell cycle were evaluated in Hepa1-6 cells *in vitro*. Briefly, Hepa1-6 cells (5×10^5 cells/well) were seeded in 6-well plates overnight. Then, 1 mL DMEM containing different formulations (free DT, free SF, Lipo/DT, Lipo/SF, or Lipo/SF/DT; DT concentration: 3 μg/mL, SF concentration: 10 μg/mL) was added to the corresponding wells and incubated for 24 h. After cells were digested for collection, they were fixed with 70% ethanol for 24 h at -4 ℃. Finally, the cells were stained with propidium iodide (PI) and detected by FCM (BD FACSCelesta). Cell cycle data were analyzed with ModFit LT software.

Deep penetration ability study of GV-Lipo/SF/DT after pre-incubated with DT. To evaluate whether DT could enhance the penetration ability of liposomes, the deep penetration ability of GV-Lipo/C6 was studied in Hepa1-6 tumor spheres. First, the Hepa1-6 tumor spheres was established using the liquid overlay method. Briefly, 30 mg/mL agarose solution was dissolved and sterilization. Agarose solution was added to 96-well plate (60 μL/well), to form agarose coated 96-well plate. A suspension containingt Matrigel matrix colloidal cells was prepared (1.0×10^4 cells/mL). The 200 μL cell suspension was added into the agarose coated 96-well plate, centrifugal (4℃, 1000 g, 10 min). After centrifugation, the 96-well plate was

placed in an incubator for culture. At the 3rd, 5th and 7th day of culture, the 100 μL medium was changed and the morphology of the Hepa1-6 tumor spheres was observed by inverted microscope. Then, the Hepa1-6 tumor spheres were co-incubated with free DT, Lipo/DT and GV-Lipo/DT (DT concentration was 3 μM) for 12 h. After co-incubation, GV-Lipo/C6 (C6 concentration was 200 ng/mL) was added and incubated. Meanwhile, Free C6 and GV-Lipo/C6 incubating with Hepa1-6 tumor spheres without DT pre-incubation were carried out. After incubation for 4 h, Hepa1-6 tumor spheres were washed with cold pH 7.4 PBS and imaged by CLSM.

Animals. Male C57BL/6 mice (5-6 weeks) were purchased from Shanghai SLAC Laboratory Animal Co., Ltd. KM mice (weight: 20 ±2 g) were purchased from the Medical Animals Experimental Center of Shandong University. All animal procedures were authorized by the Laboratory Animal Ethical and Welfare Committee of Cheeloo College of Medicine, Shandong University (Approval no. 19030).

In vivo CTC cluster dissociation assay. To test whether DT could dissociate CTC clusters *in vivo*, GFP-Hepa1-6 cells were pre-incubated with free DT or Lipo/DT (DT concentration: 3 μM) *in vitro* for 48 h. Then, single GFP Hepa1-6-luc cells (H-SL), GFP Hepa1-6-luc clusters (H-CL), free DT-pre-incubated H-CL or Lipo/DT-pre-incubated H-CL were *i.v.* injected into mice. The mice were monitored by bioluminescence imaging after *i.v.* injection of a luciferase substrate solution on days 0, 7 and 14 after cell injection. The lungs were excised and stained with H&E on the 14th day.

In vivo imaging and biodistribution. The *in vivo* targeting ability of GV-Lipo/IR780 was evaluated by a NIRF imaging and biodistribution experiment. H22 cells (1×10^6) were injected into the right axilla of KM mice to establish a tumor model. The near infrared dye IR780 iodide was used as the imaging indicator. When the tumor volume reached approximately 300 mm^3, free IR780, Lip/IR780, G-Lipo/IR780, V-Lipo/IR780 or GV-Lipo/IR780 (IR780 dose: 1 mg/kg) was *i.v.* injected into the mice. After administration of isoflurane anesthesia, the mice

were observed using a real-time NIRF detector (Caliper Life Sciences, USA) at each pre-set time point. The mice were sacrificed at 24 h after injection, and the organs and tumors were harvested for further *ex vivo* imaging evaluation.

In vivo CTC elimination assay. The ability of GV-Lipo/SF/DT to clear CTCs *in vivo* was evaluated in C67BL/6 mice *i.v.* injected with GFP Hepa1-6-luc cells. The mice were randomly separated into ten groups, and GFP Hepa1-6-luc cells (8×10^6) were *i.v.* injected into the mice, followed by treatment with different formulations (NS, free DT, free SF, Lipo/DT, Lipo/SF, V-Lipo, Lipo/SF/DT, G-Lipo/SF/DT, V-Lipo/SF/DT or GV-Lipo/SF/DT; 10 mg/kg SF, 1.0 mg/kg DT). Twenty-four hours after treatment, whole blood was collected retro-orbitally, and erythrocyte lysis buffer was added for 10 min to remove red blood cells. The collected cells, including CTCs, were separated and quantified by FCM. Mice injected without GFP Hepa1-6-luc cells were used as the negative control. GFP Hepa1-6-luc cells were used as the positive control. Relative elimination efficiency (%) was calculated using the following equation:

$$\text{Relative elimination efficiency (\%)} = \left(\frac{N_{\text{positive control}}-N_{\text{sample}}}{N_{\text{positive control}}-A_{\text{negative control}}}\right) \times 100\% \qquad (5)$$

where $N_{\text{positive control}}$ represents the CTC number of positive control group, N_{sample} represents the CTC number of treatment group, and $N_{\text{negative control}}$ represents the CTC number of negative control group.

Antitumor efficacy in a H22-bearing tumor model. An H22-bearing tumor model was established with male KM mice and used to evaluate antitumor efficacy *in vivo*. The H22 tumor-bearing mice were randomly separated into ten groups (NS, free DT, free SF, Lipo/DT, Lipo/SF, V-Lipo, Lipo/SF/DT, G-Lipo/SF/DT, V-Lipo/SF/DT and GV-Lipo/SF/DT, n=6) when the tumor volume reached 100 mm^3. The mice received intravenous treatment every 3 days with the different formulations (10 mg/kg SF, 1.0 mg/kg DT), and body weight and tumor volume were measured every 2 days (total treatment duration: 21 days). After treatment,

the mice were sacrificed. Tumor tissues were weighed. The cell proliferation and apoptosis in tumor tissues were studied by H&E staining, Ki67 staining and TUNEL staining. H&E staining of main organs was performed to evaluate the preliminary safety of the different formulations.

Antitumor efficacy in orthotopic HCC models. A GFP Hepa1-6-luc orthotopic tumor model was established with male C57BL/6 mice to further evaluate the antitumor efficacy of GV-Lipo/SF/DT *in vivo*. Orthotopic HCC mouse models were established as shown in Figure SS12. (a) Mice were anesthetized and disinfected. (b) For the surgical procedure, the abdominal cavity was cut open from the midline. (c) GFP Hepa1-6-luc cells (4×10^6) were injected slowly into the left lobe of the liver. (d) The injection site was sealed. (e) The mice were sutured and disinfected. Five days after surgery, the orthotopic tumor model mice were randomly separated into ten groups as described in the "Antitumor efficacy of GV-Lipo/SF/DT in H22-bearing tumor model" subsection. The mice were *i.v.* treated every 3 days with the different formulations (10 mg/kg SF and 1.0 mg/kg DT). The mice were observed with a bioluminescence signal detector every 2 days. Body weight was measured every 2 days (total treatment duration: 12 days). After treatment, the liver was harvested, photographed and stained with H&E for further analysis. Other organs were stained with H&E to evaluate preliminary safety.

Efficacy against in vivo tumor recurrence. The anti-recurrence effect of GV-Lipo/SF/DT was evaluated. An HCC tumor recurrence model was established with H22 tumor-bearing male KM mice. Seven days after H22 tumor inoculation, the tumors in mice were surgically removed under anesthesia. Then, the HCC tumor recurrence model mice were randomly grouped and treated as described in the "Antitumor efficacy of GV-Lipo/SF/DT in H22-bearing tumor model" subsection. The mice were photographed on day -1, day 1, day 10 and day 20. Two-month survival curves were recorded after treatment.

In vivo pulmonary metastasis inhibition study. The inhibitory effect of GV-Lipo/SF/DT on pulmonary metastasis *in vivo* was evaluated in KM mice *i.v.* injected with H22 cells. H22 cells (8×10^6) were *i.v.* injected into mice, which were then treated with different formulations as described in the "Antitumor efficacy of GV-Lipo/SF/DT in H22-bearing tumor model" subsection. The lungs were collected, imaged, and stained with H&E on the 21st day. An immunofluorescence assay was also conducted to characterize whether anti-VCAM1 mAb would effect the number of neutrophils in pulmonary metastatic nodules. The sections of lung were stained as described in "Prevention of CTC-neutrophil cluster formation in vitro". The number of pulmonary metastasis nodules was counted. Inhibition rate (%) on pulmonary metastasis was calculated using the following equation:

$$\text{Inhibition rate (\%)} = \left(\frac{N_{ns} - N_{sample}}{N_{NS}}\right) \times 100\% \ \ (\%) \qquad (6)$$

where N_{sample} represents the number of pulmonary metastasis nodules after treated with different samples, N_{NS} represents the number of pulmonary metastasis nodules of NS group.

Hemolysis assay. Samples were mixed with a red blood cell suspension (2%) and incubated at 37 °C. After centrifugation, the absorbance of hemoglobin was determined by a UV-vis spectrophotometer (576 nm). The hemolysis rates of different samples were calculated.

$$\text{Hemolysis ratio (HR \%)} = (A_x - A_0)/(A_1 - A_0) \times 100\% \qquad (7)$$

A_x, A_1, and A_0 represent the absorbance of the samples, positive control, and negative control, respectively.

Statistical analysis. Statistical analyses were calculated with GraphPad Prism 6. Student's t-tests were used for comparisons between two groups according to the data distribution. One-way analysis of variance (ANOVA) was used for comparisons of three or more groups. $p < 0.05$ was considered to indicate a significant difference.

Part B: Supplementary figures and tables

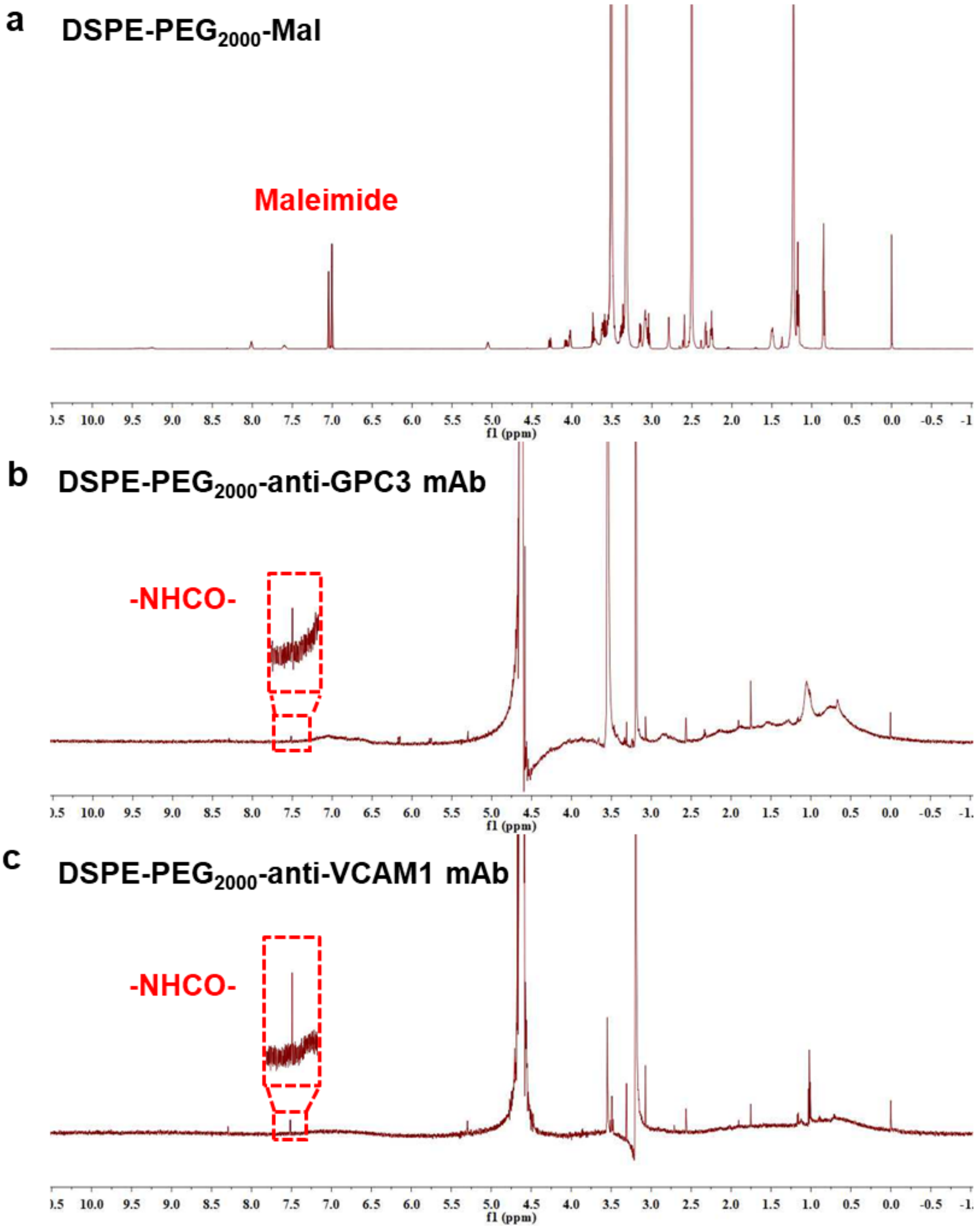

Figure S1. Characterization of DSPE-PEG$_{2000}$-anti-GPC3 mAb and DSPE-PEG$_{2000}$-anti-VCAM1 mAb. a) ^{1}H NMR spectra of DSPE-PEG$_{2000}$-Mal. b) ^{1}H NMR spectra of DSPE-PEG$_{2000}$-anti-GPC3 mAb. c) ^{1}H NMR spectra of DSPE-PEG$_{2000}$-anti-VCAM1 mAb.

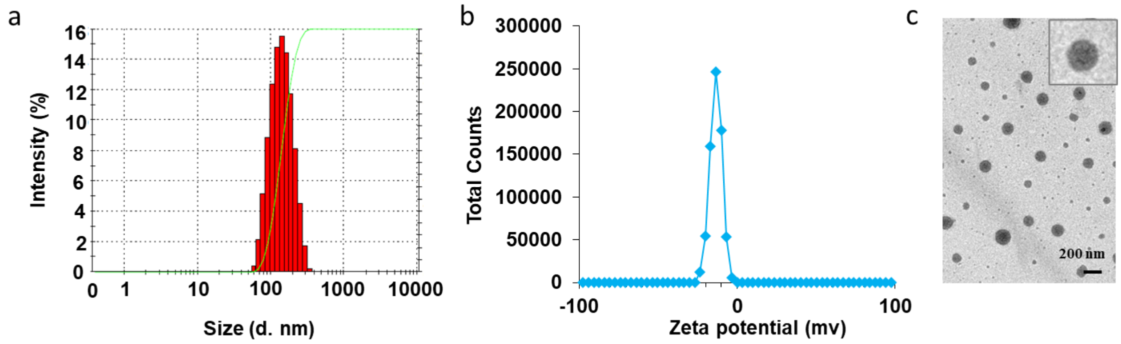

Figure S2. The characterization of Lipo/SF/DT. a) Particle size, b) zeta potential and c) transmission electron microscopy (TEM) imaging of Lipo/SF/DT.

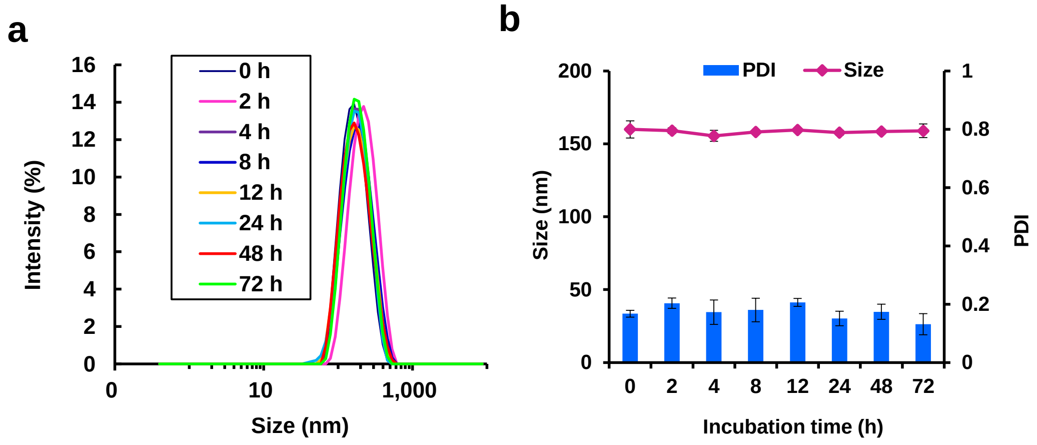

Figure S3. The plasma stability of GV-Lipo/SF/DT in 20% plasma over 72 h.

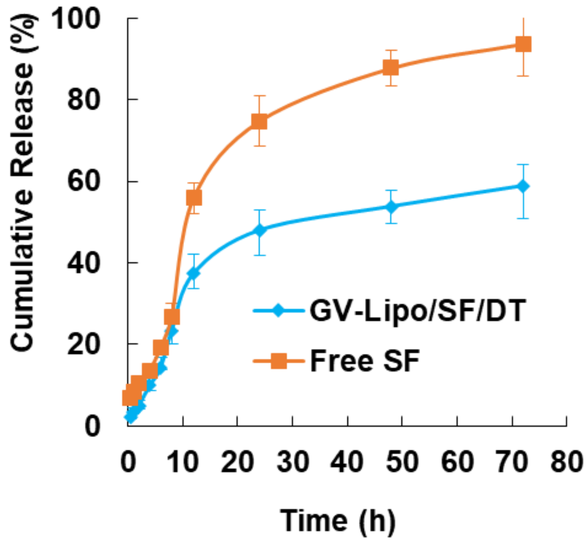

Figure S4. The cumulative release profile of SF from free SF and GV-Lipo/SF/DT in PBS buffer (pH 7.4) (n=3).

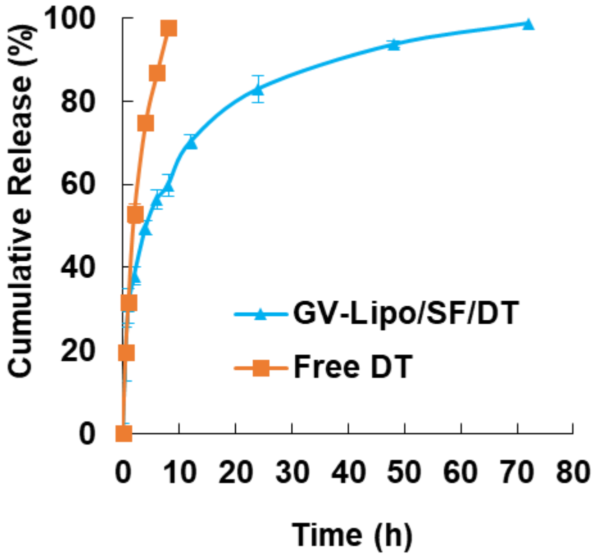

Figure S5. The cumulative release profile of DT from free DT and GV-Lipo/SF/DT in PBS buffer (pH 7.4) (n=3).

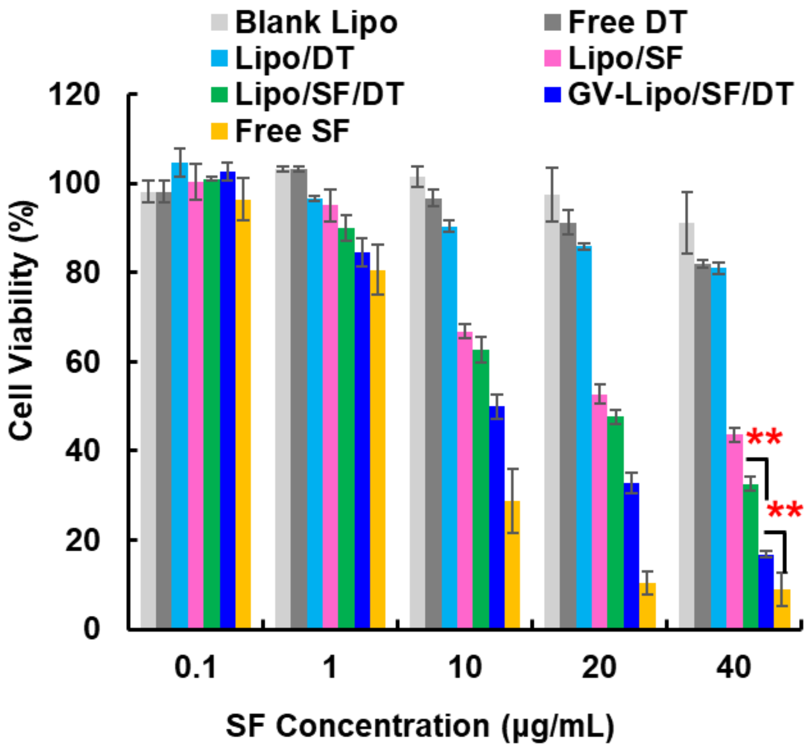

Figure S6. *In vitro* cytotoxic effects of GV-Lipo/SF/DT on Hepa1-6 cells. Cytotoxicity of blank Lipo, free DT, Lipo/DT, free SF, Lipo/SF, Lipo/SF/DT and GV-Lipo/SF/DT to Hepa1-6 cells after 48 h. n=3; ***p*<0.01.

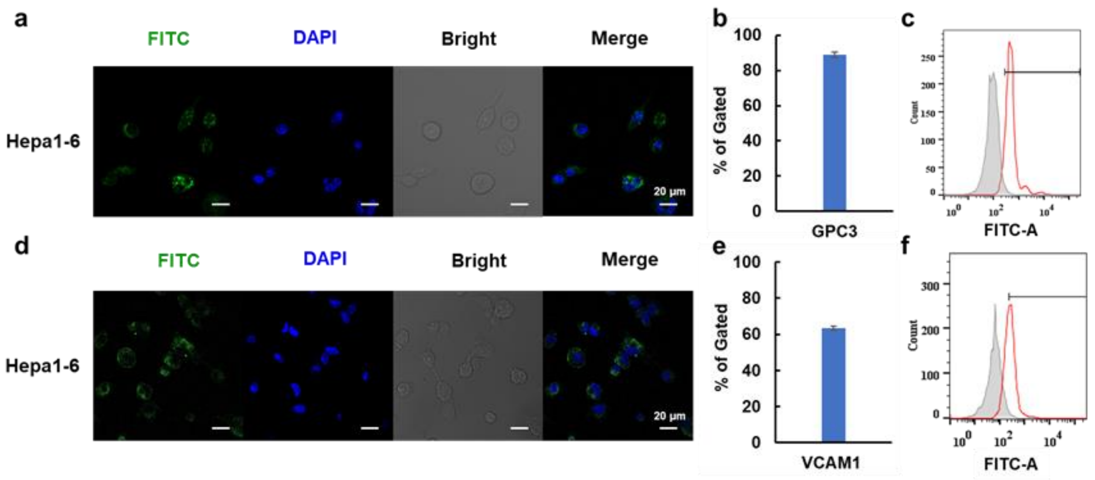

Figure S7. The expression of GPC3 and VCAM1 in Hepa1-6 cells was evaluated by CLSM and FCM. a) CLSM images, b) % of gated and c) FCM analysis of GPC3 expression in Hepa1-6 cells. d) CLSM images, e) % of gated and f) FCM analysis of VCAM1 expression in Hepa1-6 cells. Scale bar: 20 µm. Data were given as mean ± SD (n = 3).

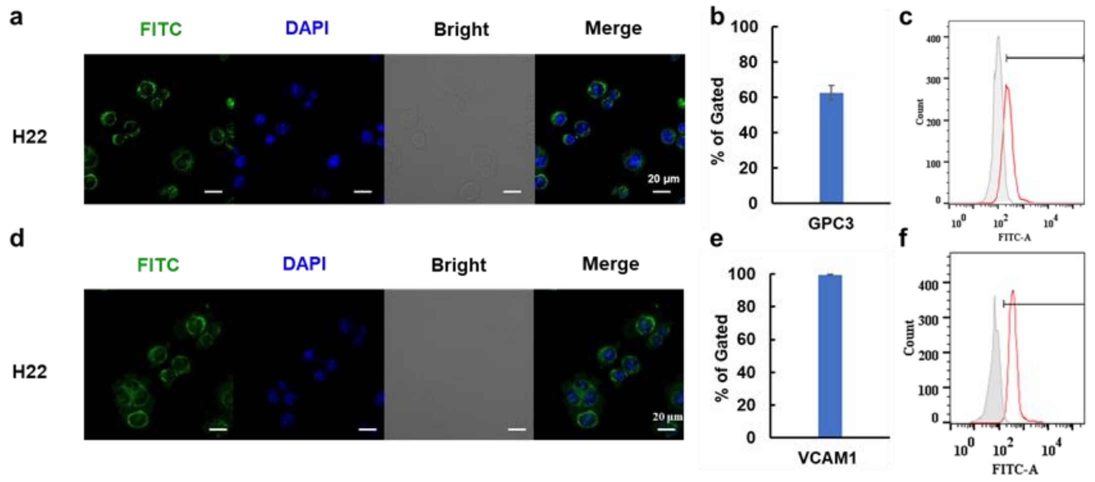

Figure S8. The expression of GPC3 and VCAM1 in H22 cells was evaluated by CLSM and FCM. a) CLSM images, b) % of gated and c) FCM analysis of GPC3 expression in H22 cells. d) CLSM images, e) % of gated and f) FCM analysis of VCAM1 expression in H22 cells. Scale bar: 20 µm. Data were given as mean ± SD (n = 3).

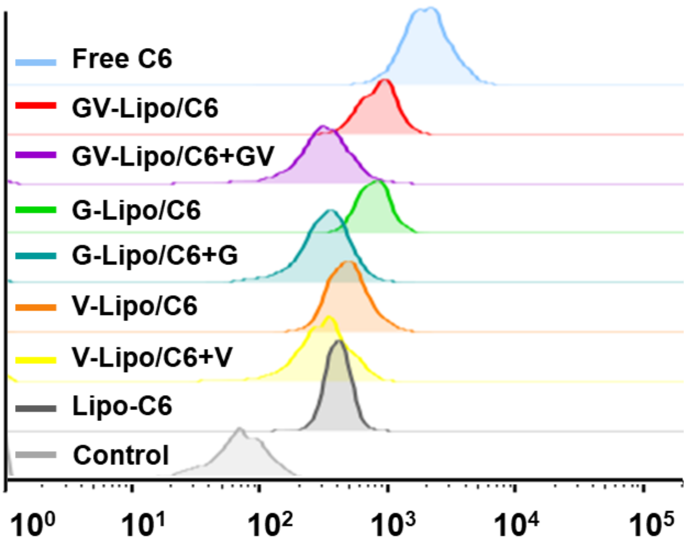

Figure S9. Cellular uptake and competitive inhibition experiments were performed with Hepa1-6 cells and free C6, Lipo/C6, G-Lipo/C6, V-Lipo/C6 or GV-Lipo/C6. FCM histogram profiles of fluorescence intensity.

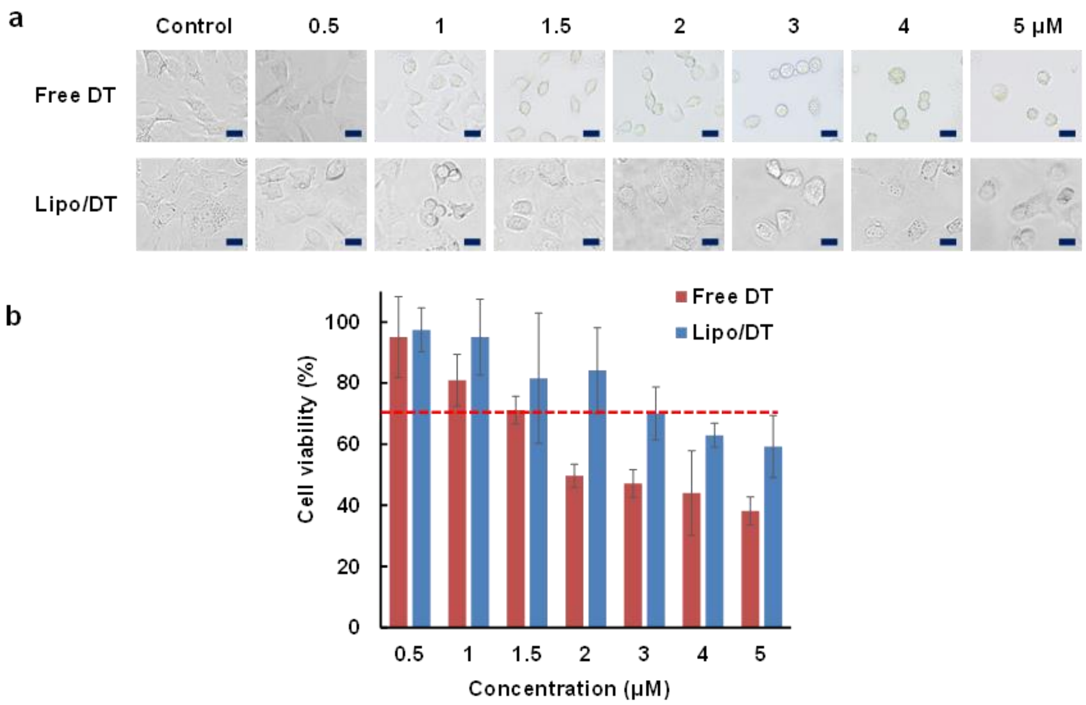

Figure S10. CTC clusters dissociation of DT was evaluated on Hepa1-6 cells *in vitro*. a) Bright field image of inverted fluorescence microscope after co-incubation with free DT and Lipo/DT, b) Cellular viability of free DT and Lipo/DT in Hepa1-6 cells for 24 h. Scale bar: 20 μm. Data were given as mean ± SD (n = 3).

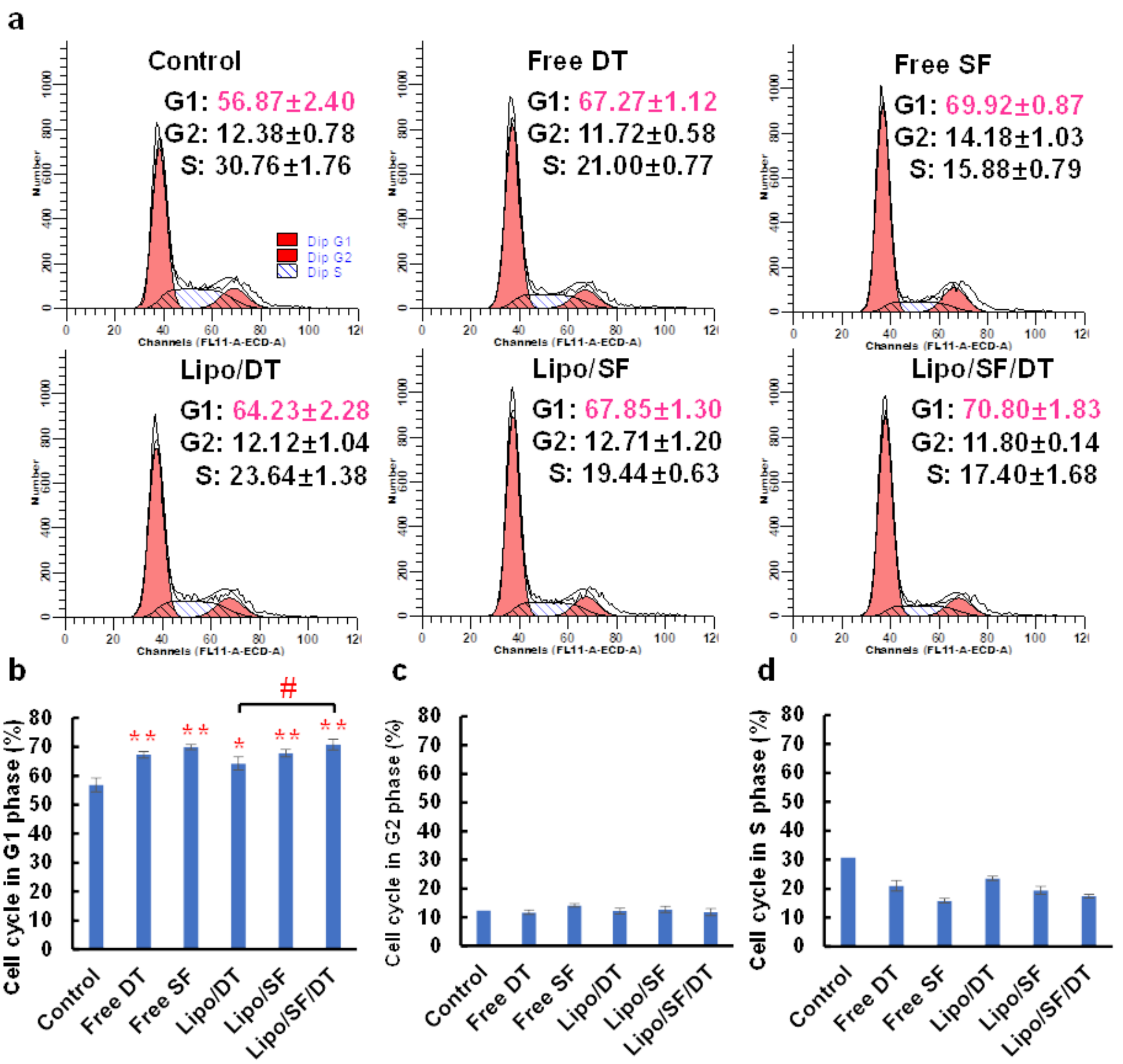

Figure S11. Cell cycle arrest was induced in the G1 phase after treated with different formulations in Hepa1-6 cells for 24 h. a) FCM histogram images of cell cycle in Hepa1-6 cells. b) Quantification percentage of cell cycle in G1 phase. n=3. c) Quantification percentage of cell cycle in G2 phase. n=3. d) Quantification percentage of cell cycle in S phase. n=3.

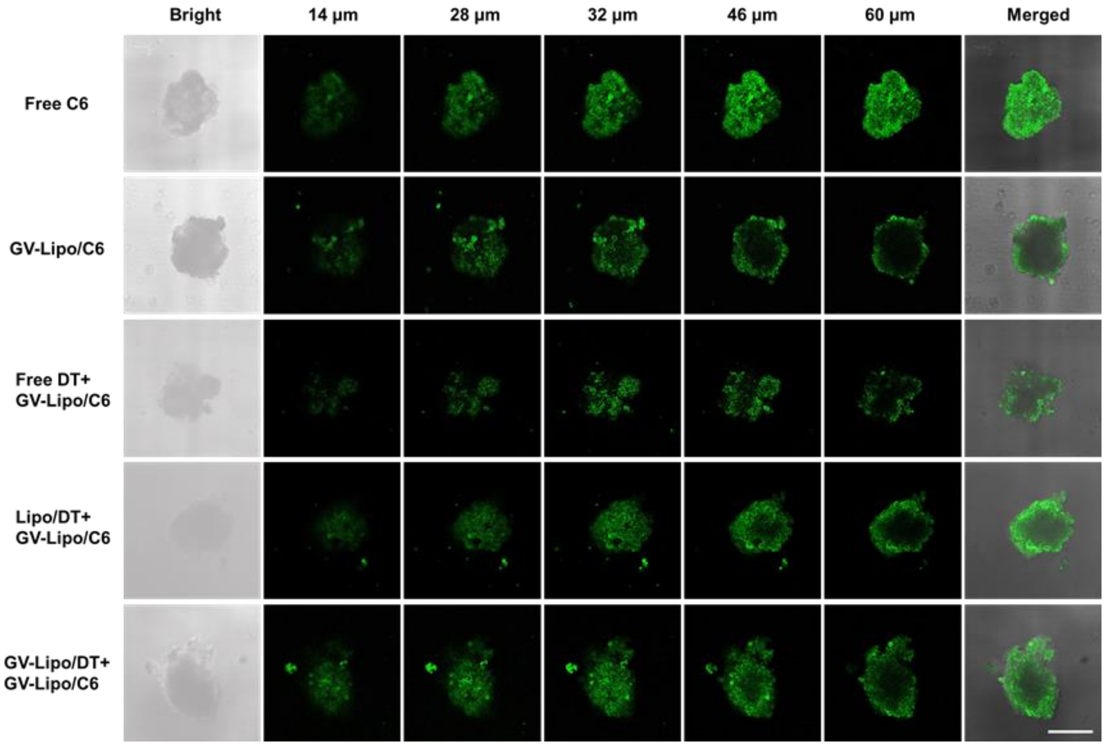

Figure S12. The tumor penetration ability of GV-Lipo/C6 was enhanced in Hepa1-6 tumor spheres after pre-incubated with free DT, Lipo/DT and GV-Lipo/DT (DT concentration 3 μM). Scale bar was 200 μm.

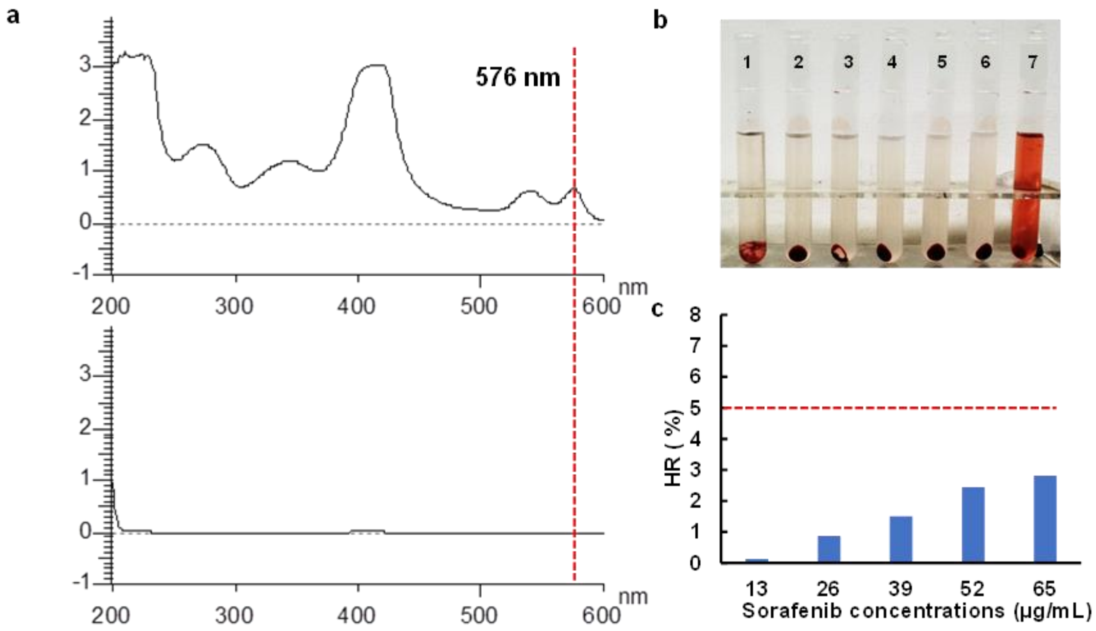

Figure S13. *In vitro* hemolysis assay of GV-Lipo/SF/DT. a) UV spectra of suspension of positive control and liposomes. b) The hemolysis image of GV-Lipo/SF/DT. c)The hemolysis ratio of GV-Lipo/SF/DT at different SF concentrations.

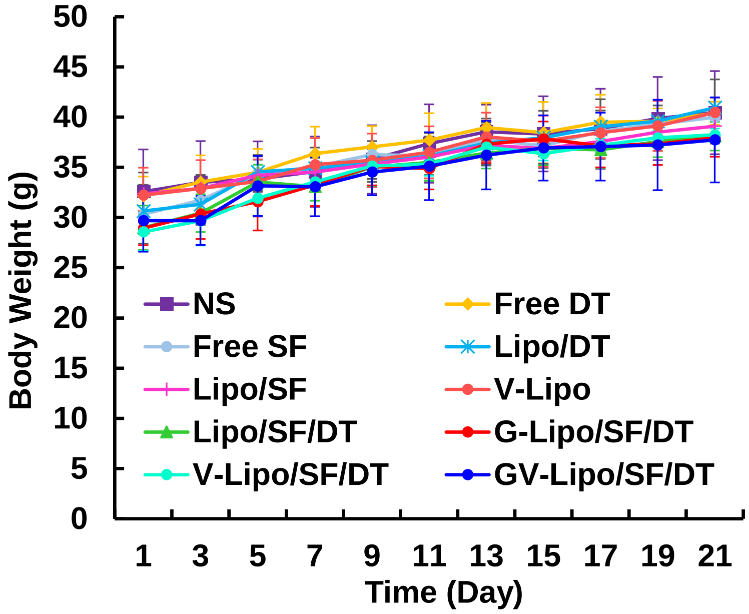

Figure S14. Body weight of H22-bearing mice treated with NS, Free DT, Free SF, Lipo/DT, Lipo/SF, V-Lipo, Lipo/SF/DT, G-Lipo/SF/DT, V-Lipo/SF/DT and GV-Lipo/SF/DT (10 mg/kg of SF, 1.0 mg/kg of DT). n = 6.

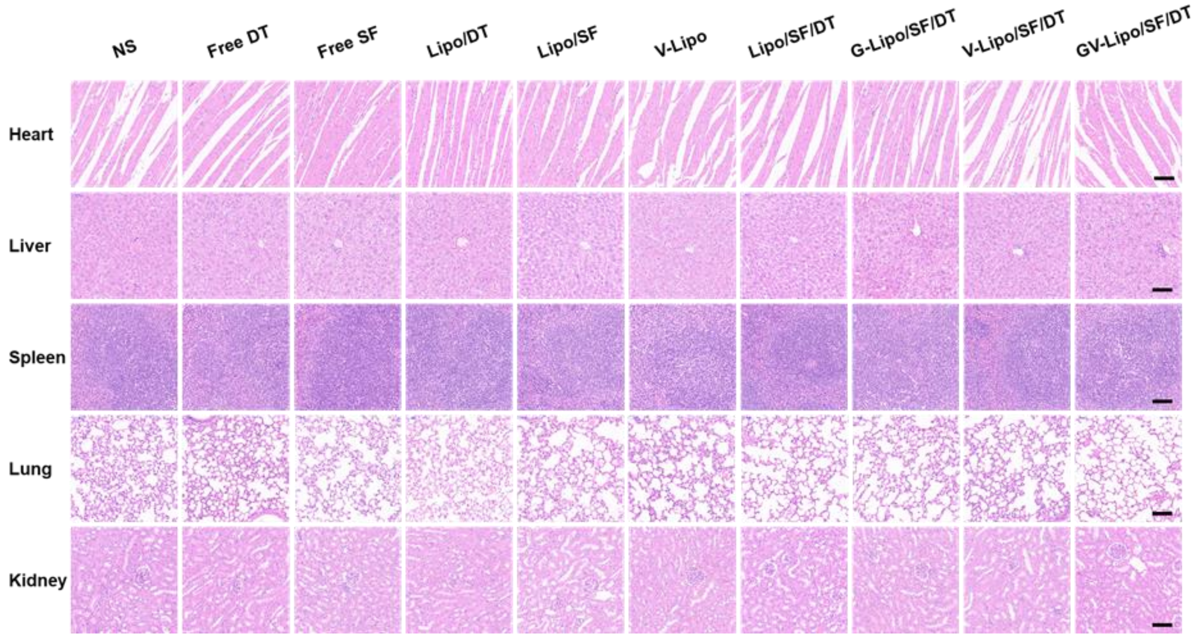

Figure S15. H&E staining of heart, liver, spleen, lung and kidneys from H22-bearing tumor model. Scale bars: 100 µm.

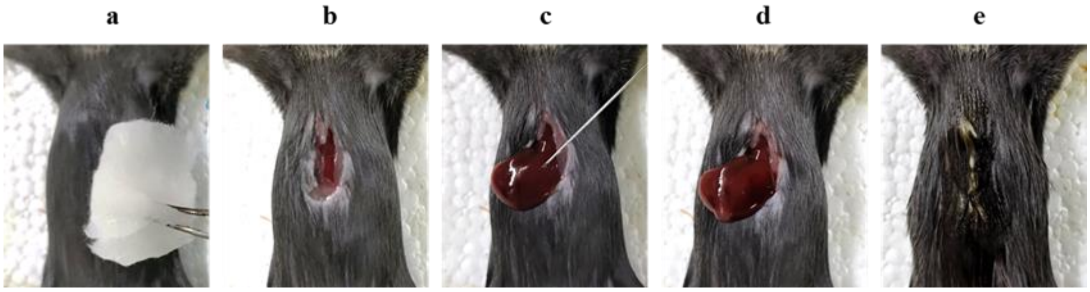

Figure S16. The establishment process of orthotopic HCC mice model. a) Anesthesia and disinfection; b) Surgery; c) Injection of GFP Hepa1-6-luc cells; d) Sealing of injection site; e) Suture and disinfection.

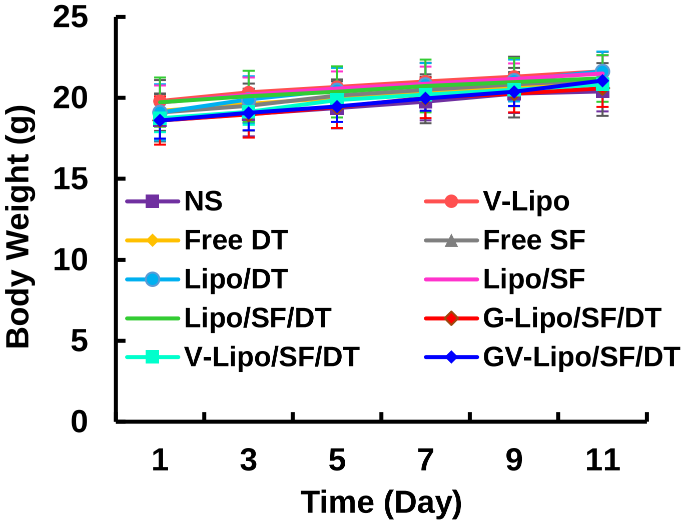

Figure S17. Body weight of orthotopic HCC mice treated with NS, Free DT, Free SF, Lipo/DT, Lipo/SF, V-Lipo, Lipo/SF/DT, G-Lipo/SF/DT, V-Lipo/SF/DT and GV-Lipo/SF/DT. n=3.

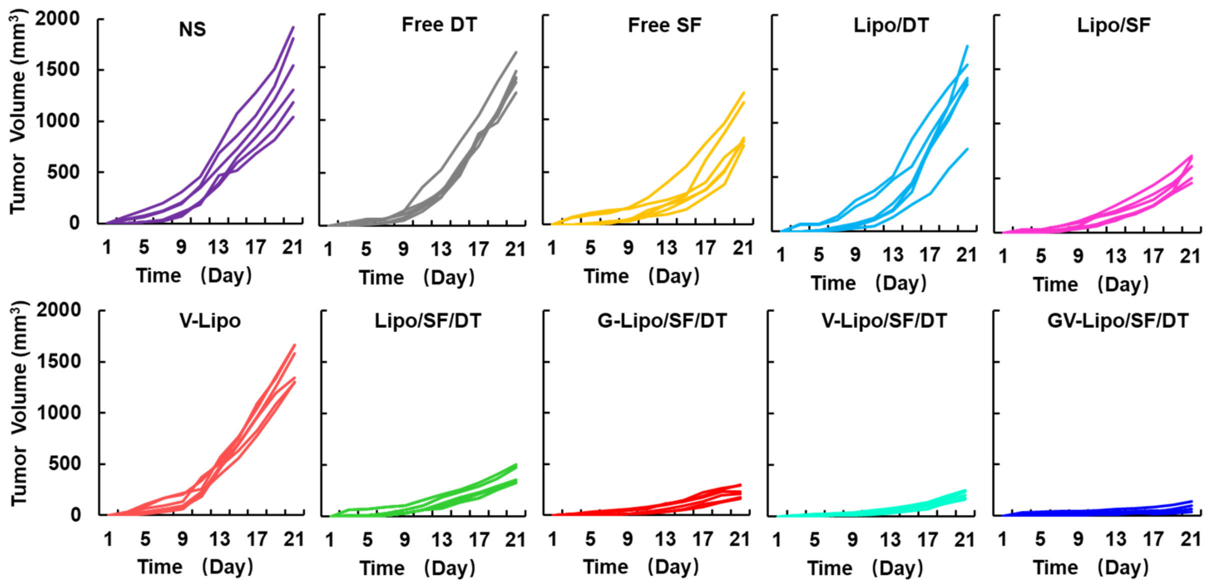

Figure S18. Individual tumor growth curves of post-surgical H22 tumor bearing mice treated with NS, Free DT, Free SF, Lipo/DT, Lipo/SF, V-Lipo, Lipo/SF/DT, G-Lipo/SF/DT, V-Lipo/SF/DT and GV-Lipo/SF/DT. n=6.

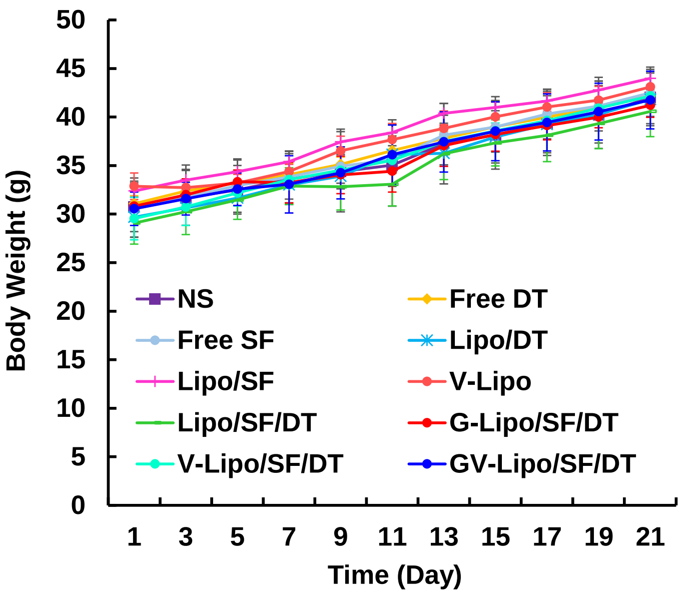

Figure S19. Body weight of post-surgical H22 tumor-bearing mice treated with NS, Free DT, Free SF, Lipo/DT, Lipo/SF, V-Lipo, Lipo/SF/DT, G-Lipo/SF/DT, V-Lipo/SF/DT and GV-Lipo/SF/DT. n=6.

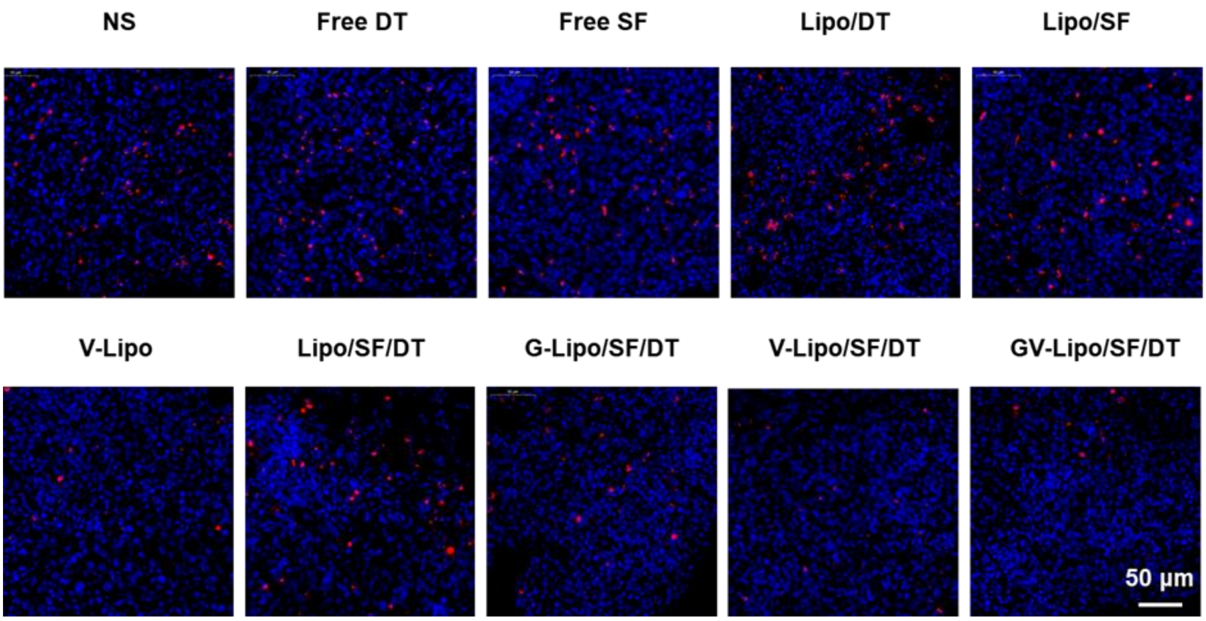

Figure S20 Representative immunofluorescence images of neutrophils colocalization in the pulmonary metastasis nodules of metastasis mice model after treated with different formulations, showing neutrophils (red), and DAPI-stained nuclei (blue). The scale bar represents 50 μm.

Table S1. Characterization of Lipo/SF/DT and GV-Lipo/SF/DT.

	Size (nm)	PDI	Zeta (mV)	SF (DL%)	SF (EE%)	DT (DL%)	DT (EE%)
Lipo/SF/DT	126.13 ±3.13	0.189 ± 0.018	-13.63 ±0.25	4.42 ±0.07	89.25 ±9.62	0.34 ±0.02	90.17 ±7.96
GV-Lipo/SF/DT	148.77 ±5.88	0.221 ± 0.019	-11.20 ±0.46	4.38 ±0.14	84.38 ±4.56	0.28 ±0.04	89.07 ±1.69

Table S2. The IC_{50} values of liposomes. $^{**}p < 0.01$ compare with GV-Lipo/SF/DT, $^{\#}p < 0.05$, $^{\#\#}p < 0.01$ compare with free SF.

	Free SF	Lipo/SF	Lipo/SF/DT	GV-Lipo/SF/DT
IC_{50} (μg/mL)	3.68±1.23	22.05±2.35$^{**, \#\#}$	11.12±1.55$^{**, \#\#}$	9.60±0.38$^{\#\#}$

Table S3. Tumor inhibition rates of different treatment groups.

Groups	Tumor inhibition rate (%)
Free DT	17.36
Free SF	52.01
Lipo/DT	22.04
Lipo/SF	63.68
V-Lipo	21.64
Lipo/SF/DT	69.10
G-Lipo/SF/DT	83.95
V-Lipo/SF/DT	82.71
GV-Lipo/SF/DT	90.28
